# Determinants of effectiveness in orthopedic ERAS: a cross-setting perspective based on the integration of nutritional status and clinical outcomes

**DOI:** 10.3389/fnut.2026.1836390

**Published:** 2026-08-25

**Authors:** Jiwei Huang, Jian Wang

**Affiliations:** The First Clinical Medical College of Lanzhou University, Lanzhou, China

**Keywords:** enhanced recovery after surgery (ERAS), nutritional status, orthopedic surgery, risk stratification, serum albumin

## Abstract

**Objective:**

This study examined factors related to the effectiveness of Enhanced Recovery After Surgery (ERAS) pathways in orthopedic patients by using data from both the National Health and Nutrition Examination Survey (NHANES) and the electronic Intensive Care Unit (eICU) database. By comparing a community-based population with critically ill hospitalized patients, we aimed to identify potential prognostic markers and setting-specific factors associated with recovery outcomes.

**Methods:**

A retrospective, multi-database comparative analysis was conducted. Participants with a history of orthopedic surgery (*n* = 2,528) were selected from NHANES cycles (2011–2018) and categorized into an “ERAS-inclined group” and a “control group” based on post-operative management characteristics. Orthopedic critically ill patients (*n* = 54,678) were selected from the eICU database (v2.0) and stratified into “survivor” and “non-survivor” groups based on hospital outcome. Data analyses included propensity score matching (PSM), multivariable logistic regression, survival analysis, machine learning models (SHAP), and decision curve analysis (DCA).

**Results:**

In the eICU cohort, the non-survivor group exhibited higher age, greater illness severity scores (APACHE and SOFA), and lower serum albumin levels (2.49 vs. 2.92 g/dL). Multivariable analysis confirmed that mechanical ventilation was the strongest factor associated with in-hospital mortality, while higher serum albumin was associated with lower mortality risk. In the matched NHANES cohort, male sex (OR = 0.45) and higher BMI (OR = 0.97) were identified as protective factors for improved long-term rehabilitation outcomes, while widowed status was a risk factor. Cross-database comparison showed that serum albumin levels were highest in the NHANES community cohort, generally around 4.0 g/dL, and lower in the eICU critically ill cohort, generally around 2.5–3.0 g/dL. This pattern suggests that albumin may be a useful marker for reflecting nutritional status and illness severity across different clinical settings. The NHANES model showed better discrimination, whereas the eICU model demonstrated modest discrimination, suggesting that the identified predictors may be useful for hypothesis generation and risk stratification, but require further validation before clinical application.

**Conclusion:**

The findings of this study indicate that the factors associated with rehabilitation in orthopedic patients may differ depending on the stage of recovery. In the acute critical phase, the need for mechanical ventilation may reflect more severe physiological stress, while hypoalbuminemia may suggest underlying nutritional depletion; both were associated with poorer clinical outcomes. In contrast, during long-term recovery, baseline BMI and marital status appeared to have predictive relevance, possibly reflecting metabolic reserve and social support. These results suggest that nutritional risk assessment and evaluation of social vulnerability should receive greater attention in orthopedic ERAS practice. However, as this study is mainly exploratory, the findings should be confirmed in future prospective research.

## 1 Introduction

The global acceleration of population aging has led to a surge in the demand for orthopedic surgeries, making the optimization of perioperative management to improve patient outcomes a focal point of clinical research (1, 2). Enhanced Recovery After Surgery (ERAS), through a series of evidence-based interventions, aims to reduce surgical stress and accelerate functional recovery (3–5). Although the application of ERAS in orthopedics has shown potential in shortening hospital stays and reducing complication rates, its efficacy exhibits significant heterogeneity across different patient populations and clinical settings (6, 7). Elective arthroplasty, spine surgery, and fracture/trauma patients differ substantially in baseline frailty, urgency, and rehabilitation tolerance, and should not be assumed to form a homogeneous ERAS population. In older orthopedic patients, frailty provides a useful way to understand this heterogeneity (8). It encompasses reduced muscle mass, impaired baseline function, cognitive vulnerability, poor nutritional reserve, and limited social support, factors that may influence both patients’ tolerance of ERAS components and their postoperative recovery. This reflects a “vulnerability paradox,” where patients most in need of ERAS support may also require a more individualized pathway because of limited physiological reserve.

This heterogeneity may be related to several gaps in current research. First, many studies have focused mainly on short-term in-hospital outcomes, while less attention has been paid to how patients’ baseline health status, such as nutritional condition and metabolic function, influences longer-term recovery after ERAS (9, 10). Second, in patients who experience major surgical stress and require intensive care, the extent to which ERAS principles remain applicable is still uncertain (11, 12). Because most previous studies have been based on a single database or a single center, it is difficult to examine these issues across different stages of care.

To overcome this limitation, this study innovatively adopts a cross-setting, multi-database integrated analysis strategy. We utilized two authoritative, highly complementary databases in parallel: NHANES and eICU. The NHANES database provides deep phenotyping data for a large community population, making it an ideal resource for assessing preoperative long-term health baselines (13, 14). The eICU database records high-intensity clinical time-series data from multicenter ICU patients, offering a unique window to dissect the relationship between perioperative acute interventions and critical outcomes (15, 16).

This study does not directly assess ERAS pathway adherence or its causal effects. Instead, it uses observational data to explore prognostic factors related to recovery and survival in orthopedic patients across different care settings. Based on these two complementary data sources, this study had three main aims. First, we compared factors associated with long-term recovery in the NHANES orthopedic cohort and short-term survival in the eICU orthopedic cohort. Second, we explored whether some markers, particularly serum albumin, showed consistent associations with outcomes across the two settings. Third, we developed and evaluated prediction models for the two cohorts, with the goal of providing preliminary evidence for more individualized risk assessment in orthopedic ERAS practice (17–20).

## 2 Research methods

### 2.1 Study design and data sources

This retrospective observational study used data from two independent public databases. The datasets were analyzed separately, and no individual-level linkage or direct patient identification was performed. All analyses followed the data-use requirements and ethical standards of the respective databases.

NHANES Database: Administered by the National Center for Health Statistics, it employs a complex, multi-stage probability sampling design. Data are collected through household interviews, mobile examination center (MEC) examinations, and laboratory tests, representing the non-institutionalized civilian population of the United States (21).

eICU Collaborative Research Database: Hosted by the MIT Laboratory for Computational Physiology, it contains de-identified clinical data from over 200 hospital ICUs across the United States.

### 2.2 Study population and grouping

NHANES Community Orthopedic Cohort: From the 2011–2018 survey cycles, participants aged ≥18 years with self-reported history of major elective or traumatic orthopedic surgery (including hip/knee arthroplasty, spinal fusion, internal fixation for long bone fractures, etc.) were selected. Latent class analysis was performed using questionnaire-based indicators of postoperative recovery, including early ambulation, multimodal analgesia, and early oral intake. The resulting class was regarded as an ERAS-inclined recovery profile, rather than a direct measure of ERAS exposure, because it may capture both ERAS-related care elements and patients’ underlying health reserve. Ultimately, 2,528 individuals were included for analysis, with 355 in the ERAS-inclined recovery profile group and 2,173 in the control profile group (Table 1).

**Table 1:** Demographic characteristics, comorbidities, and laboratory parameters of NHANES participants stratified by ERAS grouping.

	Control	ERAS_group	*p*
n	2,173	355	
Gender = Male (%)	1,198 (55.1)	67 (18.9)	<0.001
Ethnicity (%)			<0.001
Mexican American	263 (12.1)	30 (8.5)	
Non-Hispanic Asian	287 (13.2)	47 (13.2)	
Non-Hispanic Black	518 (23.8)	61 (17.2)	
Non-Hispanic White	791 (36.4)	173 (48.7)	
Other Hispanic	219 (10.1)	33 (9.3)	
Other Race	95 (4.4)	11 (3.1)	
JointReplacement = Yes (%)	0 (0.0)	55 (15.5)	<0.001
FractureHistory = Yes (%)	0 (0.0)	317 (89.3)	<0.001
PhysicalActivity = Yes (%)	439 (20.2)	56 (15.8)	0.061
Smoking = Yes (%)	780 (35.9)	127 (35.8)	1.000
Drinking = Yes (%)	821 (37.8)	136 (38.3)	0.896
Education (%)			0.203
High	675 (31.1)	94 (26.5)	
Low	639 (29.4)	108 (30.4)	
Medium	859 (39.5)	153 (43.1)	
Marital_status (%)			0.002
Divorced	334 (15.4)	40 (11.3)	
Married	1,076 (49.5)	189 (53.2)	
Single	444 (20.4)	54 (15.2)	
Widowed	319 (14.7)	72 (20.3)	
Age_Group (%)			<0.001
<65	1,232 (56.7)	119 (33.5)	
≥80	215 (9.9)	74 (20.8)	
65–79	726 (33.4)	162 (45.6)	
BMI_Group (%)			0.002
Normal	463 (21.3)	99 (27.9)	
Obese	932 (42.9)	118 (33.2)	
Overweight	755 (34.7)	131 (36.9)	
Underweight	23 (1.1)	7 (2.0)	
Age [mean (SD)]	64.15 (8.95)	69.05 (8.84)	<0.001
BMI [mean (SD)]	29.83 (6.52)	28.73 (6.87)	0.004
Waist [mean (SD)]	103.41 (15.53)	99.95 (15.70)	<0.001
Albumin [mean (SD)]	4.03 (0.31)	3.96 (0.34)	<0.001
Creatinine [mean (SD)]	0.97 (0.52)	0.90 (0.44)	0.025
Glucose [mean (SD)]	110.57 (44.30)	103.23 (30.33)	0.003
Bilirubin [mean (SD)]	0.49 (0.28)	0.43 (0.22)	<0.001
Vitamin D [mean (SD)]	21.87 (8.01)	21.88 (8.01)	0.984

eICU Orthopedic Critical Care Cohort: Adult patients with primary diagnoses or procedure codes (based on ICD-9-CM) belonging to the musculoskeletal system and postoperative care categories, and with an ICU length of stay exceeding 24 h were selected. Patients were stratified into survivor and non-survivor groups according to hospital discharge status. The primary outcome for the eICU cohort was in-hospital mortality during the index hospitalization, rather than fixed-time 28-day or 180-day mortality (Table 2).

**Table 2:** Definition and sources of core study variables.

	level	Alive	Dead	*p*
n		48,504	6,174	
Age [mean (SD)]		61.79 (17.13)	68.19 (14.75)	<0.001
Gender (%)	Female	22,229 (45.8)	2,786 (45.1)	0.302
	Male	26,275 (54.2)	3,388 (54.9)	
Ethnicity (%)	Black	5,239 (10.8)	593 (9.6)	0.001
	Hispanic	1866 (3.8)	202 (3.3)	
	Other	3,105 (6.4)	359 (5.8)	
	Unknown	717 (1.5)	102 (1.7)	
	White	37,577 (77.5)	4,918 (79.7)	
BMI [mean (SD)]		29.05 (8.37)	28.52 (8.78)	<0.001
Age_Group (%)	<65	25,360 (52.3)	2,269 (36.8)	<0.001
	> = 80	7,954 (16.4)	1,570 (25.4)	
	65–79	15,190 (31.3)	2,335 (37.8)	
BMI_Group (%)	Normal	14,480 (29.9)	2011 (32.6)	<0.001
	Obese	17,807 (36.7)	2078 (33.7)	
	Overweight	14,071 (29.0)	1,674 (27.1)	
	Underweight	2,146 (4.4)	411 (6.7)	
ICU_Type (%)	Burn ICU	1,111 (2.3)	120 (1.9)	<0.001
	Cardiac ICU	2,381 (4.9)	305 (4.9)	
	Medical ICU	28,358 (58.5)	3,489 (56.5)	
	Mixed ICU	3,432 (7.1)	383 (6.2)	
	Neuro ICU	3,219 (6.6)	459 (7.4)	
	Other ICU	1754 (3.6)	197 (3.2)	
	Surgical ICU	4,505 (9.3)	790 (12.8)	
	Trauma ICU	3,744 (7.7)	431 (7.0)	
Admission_Type (%)	Elective	44,187 (91.1)	5,419 (87.8)	<0.001
	Emergency	2,175 (4.5)	303 (4.9)	
	Transfer	2,142 (4.4)	452 (7.3)	
Hospital_LOS [mean (SD)]		8.11 (9.78)	5.67 (5.65)	<0.001
ICU_LOS [mean (SD)]		3.40 (4.86)	4.30 (4.47)	<0.001
APACHE_Score [mean (SD)]		59.43 (24.08)	97.50 (31.54)	<0.001
SOFA_Score [mean (SD)]		3.25 (2.65)	6.38 (3.40)	<0.001
GCS [mean (SD)]		12.92 (3.36)	9.63 (4.80)	<0.001
Intubation (%)	No	40,900 (84.3)	3,655 (59.2)	<0.001
	Yes	7,604 (15.7)	2,519 (40.8)	
Ventilation (%)	No	36,323 (74.9)	2,666 (43.2)	<0.001
	Yes	12,181 (25.1)	3,508 (56.8)	
Diabetes (%)	No	36,470 (75.2)	4,796 (77.7)	<0.001
	Yes	12,034 (24.8)	1,378 (22.3)	
Creatinine [mean (SD)]		1.66 (1.91)	2.21 (1.75)	<0.001
Albumin [mean (SD)]		2.92 (0.68)	2.49 (0.70)	<0.001
Glucose [mean (SD)]		162.64 (103.38)	182.17 (116.73)	<0.001

### 2.3 Study variables

For the NHANES cohort, the primary outcome was whether a participant belonged to the ERAS-inclined postoperative recovery profile. This profile was derived using latent class analysis based on questionnaire indicators of postoperative recovery, including early ambulation, multimodal analgesia, and early oral intake. The outcome was analyzed as a binary variable, comparing the ERAS-inclined recovery profile with the control profile. Variables were chosen according to clinical relevance and previous evidence on perioperative outcomes. Serum albumin was included because it reflects nutritional reserve and inflammatory status. BMI was used as a simple indicator of metabolic reserve and body composition, while marital status was included to capture psychosocial support and social vulnerability.

### 2.4 Statistical analysis

All analyses were performed using R (version 4.3.0) and Python (version 3.9), with a two-sided significance level of *α* = 0.05.

Baseline Description and Comparison: Continuous variables were described as mean ± standard deviation or median (interquartile range) based on distribution, with group comparisons using t-tests or Mann–Whitney U tests. Categorical variables were described as frequency (percentage), with group comparisons using chi-square tests. Baseline characteristics for the NHANES and eICU cohorts are summarized in Tables 1, 2, respectively.

**Figure 1:**
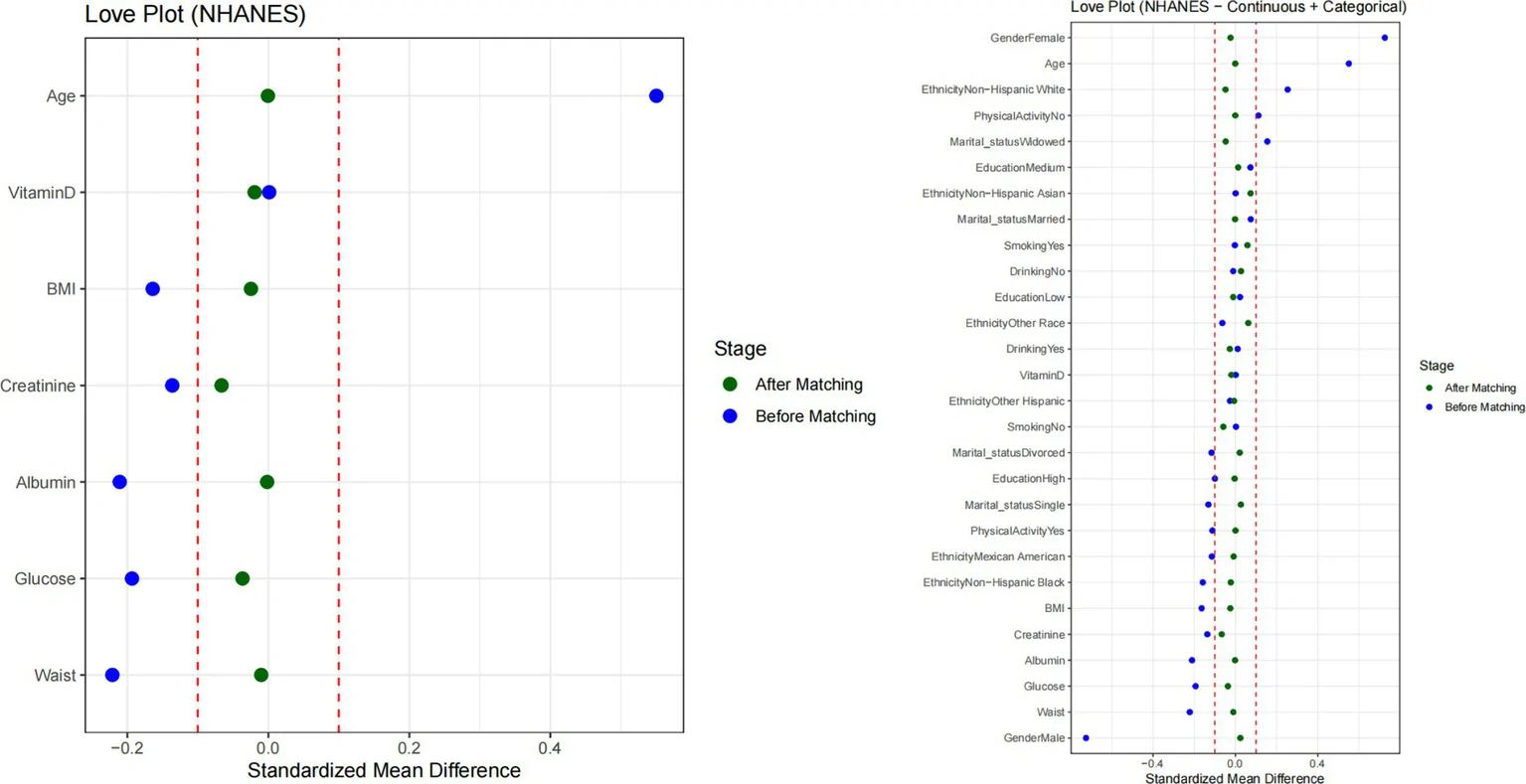
Love plot illustrating the standardized mean differences of covariates before and after propensity score matching. This figure visually demonstrates the effectiveness of propensity score matching (PSM) in reducing imbalances in baseline characteristics. The blue dots represent standardized mean differences before matching, whereas the green dots represent those after matching. Differences before matching: Prior to matching, several covariates, such as male sex, age, and non-Hispanic White ethnicity, exhibited relatively large absolute standardized mean differences (SMDs), with values substantially deviating from zero, indicating considerable baseline imbalance between the two groups. Balance after matching: Following PSM, the SMDs of nearly all covariates converged markedly toward zero and fell within a narrow range (within ±0.1). This indicates that good covariate balance was achieved between the two groups after matching in terms of demographic characteristics, lifestyle factors, and laboratory parameters, thereby effectively reducing the potential influence of confounding factors.

Addressing Confounding and Selection Bias (for NHANES cohort): Because the present analysis was intended as an exploratory matched comparison rather than a national prevalence estimate, survey weights were not incorporated; therefore, the results should not be interpreted as nationally representative. Due to significant selection bias in the ERAS grouping (Table 1), propensity score matching (PSM) was employed for adjustment. A generalized boosted model (GBM) was fitted with ERAS grouping as the dependent variable, incorporating all baseline variables from Table 1 to estimate propensity scores. Subsequent 1:1 nearest neighbor matching was performed with a caliper width of 0.02. Matching effectiveness was evaluated via standardized mean differences (SMD) and visualized with a Love Plot (Figure 1); SMD < 0.1 indicated good covariate balance. All subsequent analyses for the NHANES cohort were conducted on the matched sample (*n* = 710). The marked differences in clinical composition suggest that the two groups may not be fully comparable, even after matching. Therefore, the matched NHANES analyses should be viewed as exploratory rather than confirmatory.

**Figure 2:**
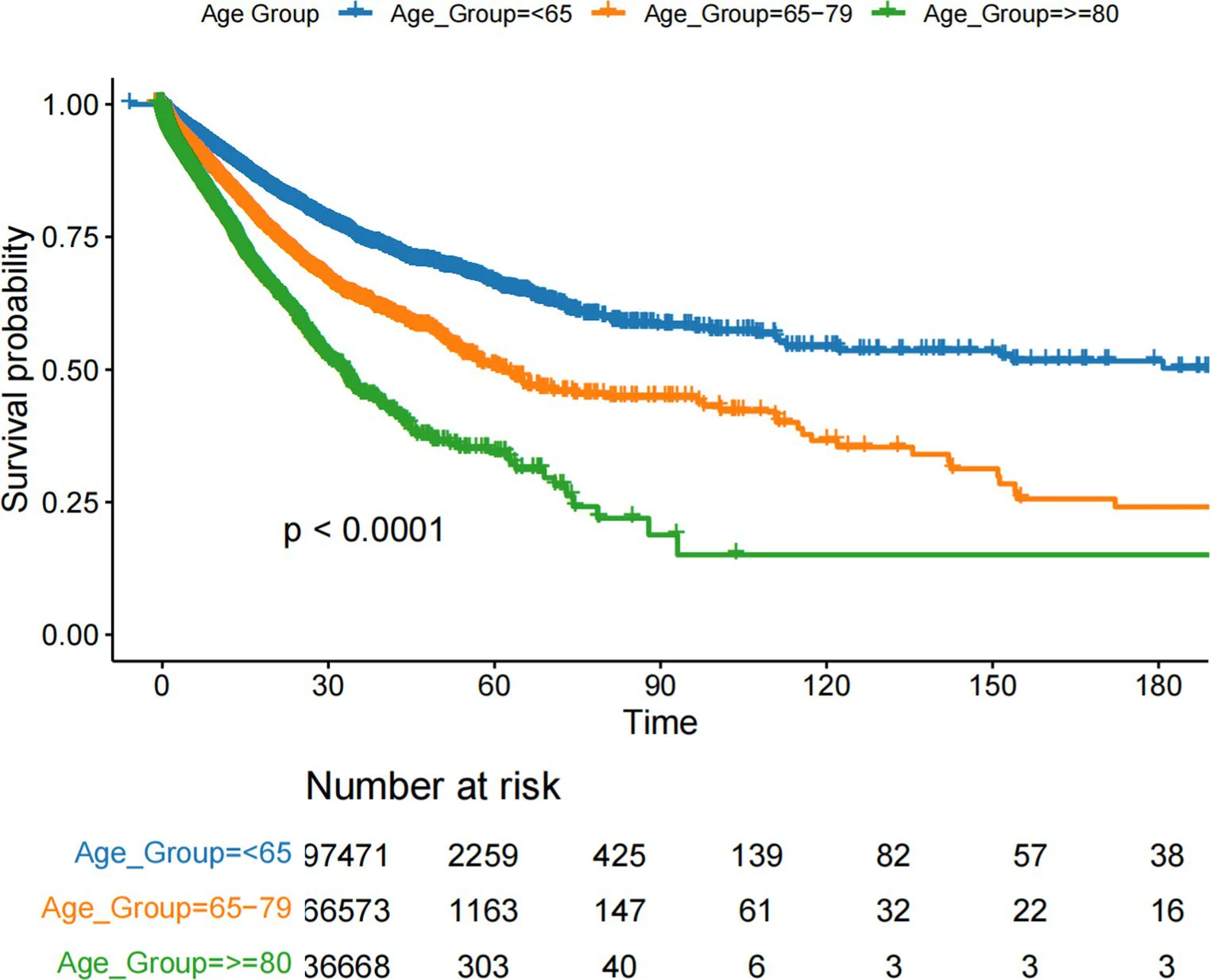
Kaplan–Meier survival curves stratified by age groups over a 180-day follow-up period. This figure illustrates the changes in survival probability over a 180-day follow-up period among patients in different age groups. Participants were categorized into three groups according to age: <65 years (blue), 65–79 years (orange), and ≥80 years (green). The log-rank test demonstrated a statistically significant difference in survival curves among the three age groups (p < 0.0001). Patients aged <65 years exhibited the highest survival probability, with the most gradual decline in the survival curve. In contrast, patients aged ≥80 years showed the most rapid and pronounced decline in survival probability, indicating that advanced age is a significant risk factor for poorer long-term prognosis among critically ill patients. The risk table presents the number of patients at risk in each age group at different time points throughout the follow-up period, showing a progressive decrease over time and further illustrating the temporal distribution of the study population.

Survival Analysis (for eICU cohort): For the eICU cohort, Kaplan–Meier curves were used to describe observed in-hospital survival from ICU admission to hospital death. Patients discharged alive were censored at hospital discharge. Because post-discharge follow-up was not available, the survival analysis was not interpreted as 28-day or 180-day mortality (Figure 2).

**Figure 3:**
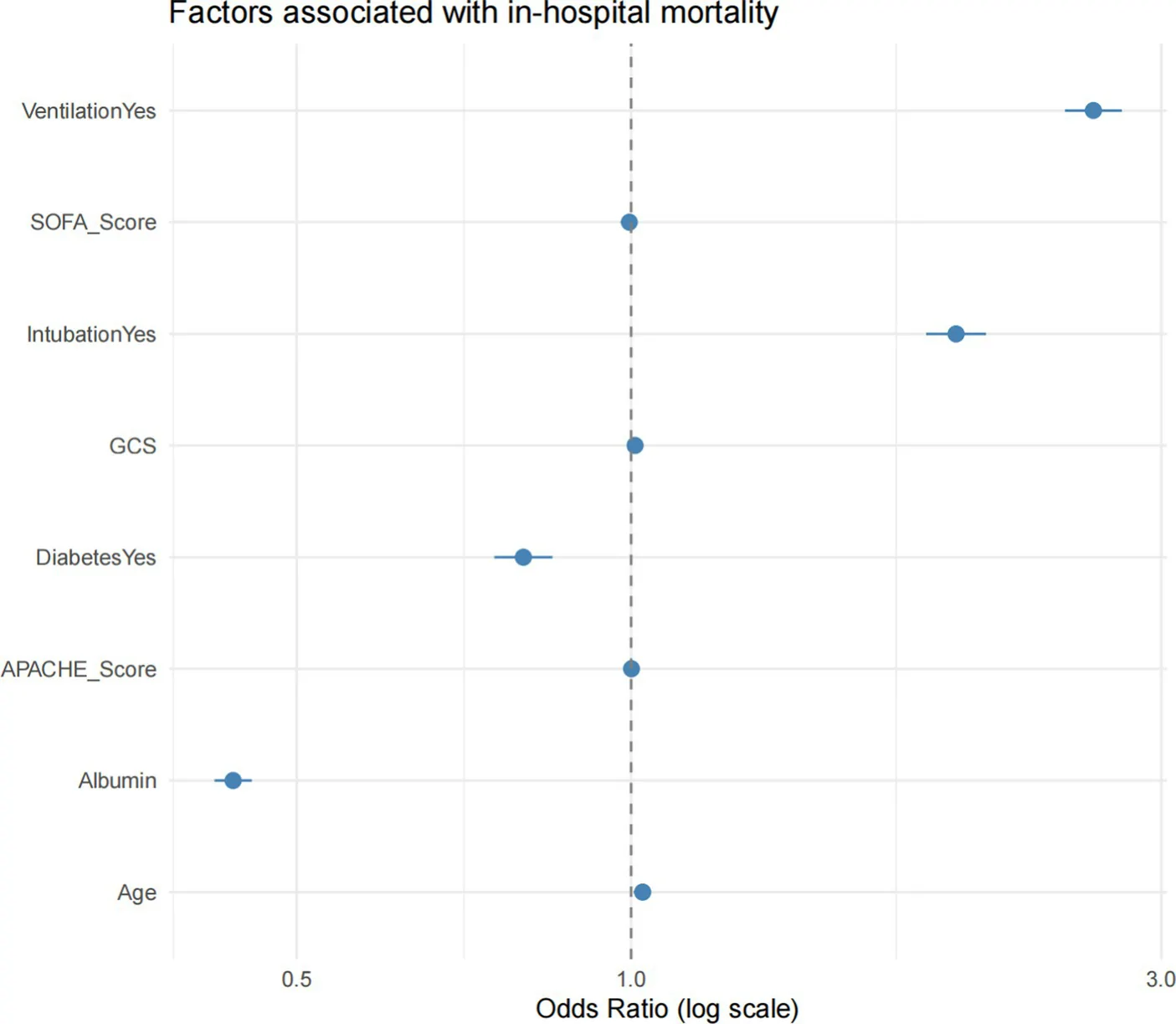
Forest plot illustrating the odds ratios of significant predictors identified by multivariable logistic regression for 28-day mortality.This figure presents the direction and magnitude of the associations between variables included in the regression model and the risk of 28-day mortality, expressed as odds ratios (ORs). Risk factors: Mechanical ventilation (Ventilation: Yes) and endotracheal intubation (Intubation: Yes) are located to the right of the vertical reference line, with ORs significantly greater than 1, indicating that the need for respiratory support is significantly associated with an increased risk of mortality. Age also appears to be a risk factor for mortality. Protective factors: The point estimates for albumin and a history of diabetes (Diabetes: Yes) are located to the left of the reference line (OR < 1). In particular, higher serum albumin levels are associated with a more favorable prognosis. Clinical severity scores: The effect estimates for SOFA, APACHE, and GCS scores are close to 1, suggesting that after adjustment for other strong predictors, such as ventilation status, their independent predictive effects are relatively modest in this multivariable model.

**Figure 4:**
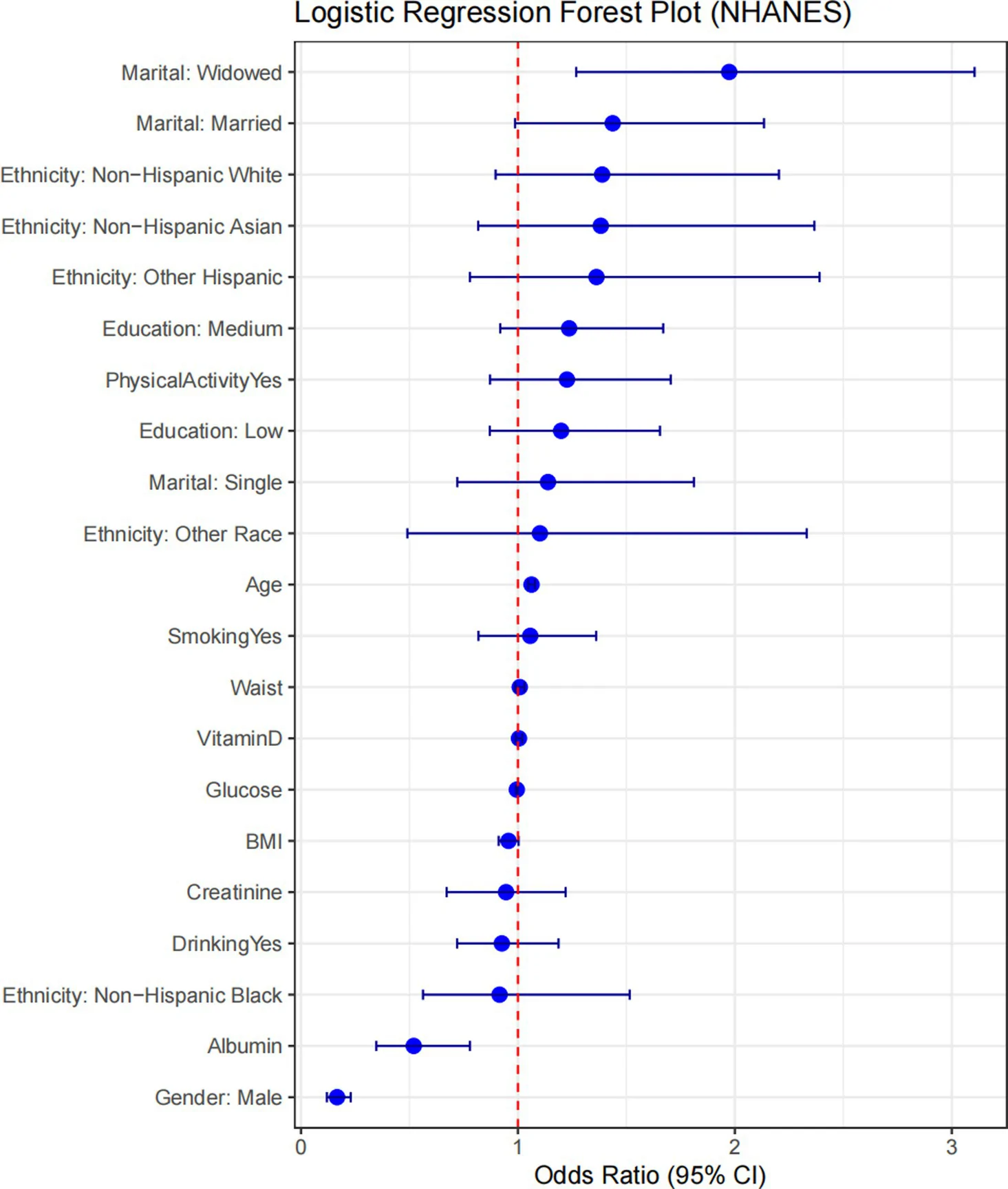
Forest plot of multivariable logistic regression analysis identifying iIndependent predictors associated with the outcome. This figure presents the odds ratios (ORs) and corresponding 95% confidence intervals (CIs) for the associations between individual variables and the outcome in the NHANES cohort. Protective factors: Male sex (Gender: Male) shows the most pronounced protective association, with an OR substantially below 1. In addition, higher albumin levels and higher BMI are associated with ORs < 1, suggesting that better nutritional reserves may be associated with more favorable outcomes. Risk factors: Widowed marital status (Marital: Widowed) is associated with a higher OR, suggesting that reduced social support may represent a potential risk factor for the outcome. Other variables: Variables such as vitamin D, glucose, and alcohol consumption (Drinking: Yes) have ORs close to 1, with their 95% CIs crossing the null value of 1.0, indicating that their independent associations with the outcome do not reach statistical significance in this multivariable model.

Prognostic Factor Identification and Model Building: Multivariable logistic regression models were constructed for the primary outcome in each cohort. Candidate variables were selected mainly according to clinical relevance, prior evidence, and data availability, rather than solely on univariate screening. Adjusted odds ratios and 95% confidence intervals are presented in forest plots for the eICU and NHANES cohorts. Given the exploratory nature of this study and the limited clinical overlap in the matched NHANES sample, model estimates should be interpreted cautiously, and further validation in independent orthopedic cohorts is required. (eICU: Figure 3, NHANES: Figure 4).

**Figure 5:**
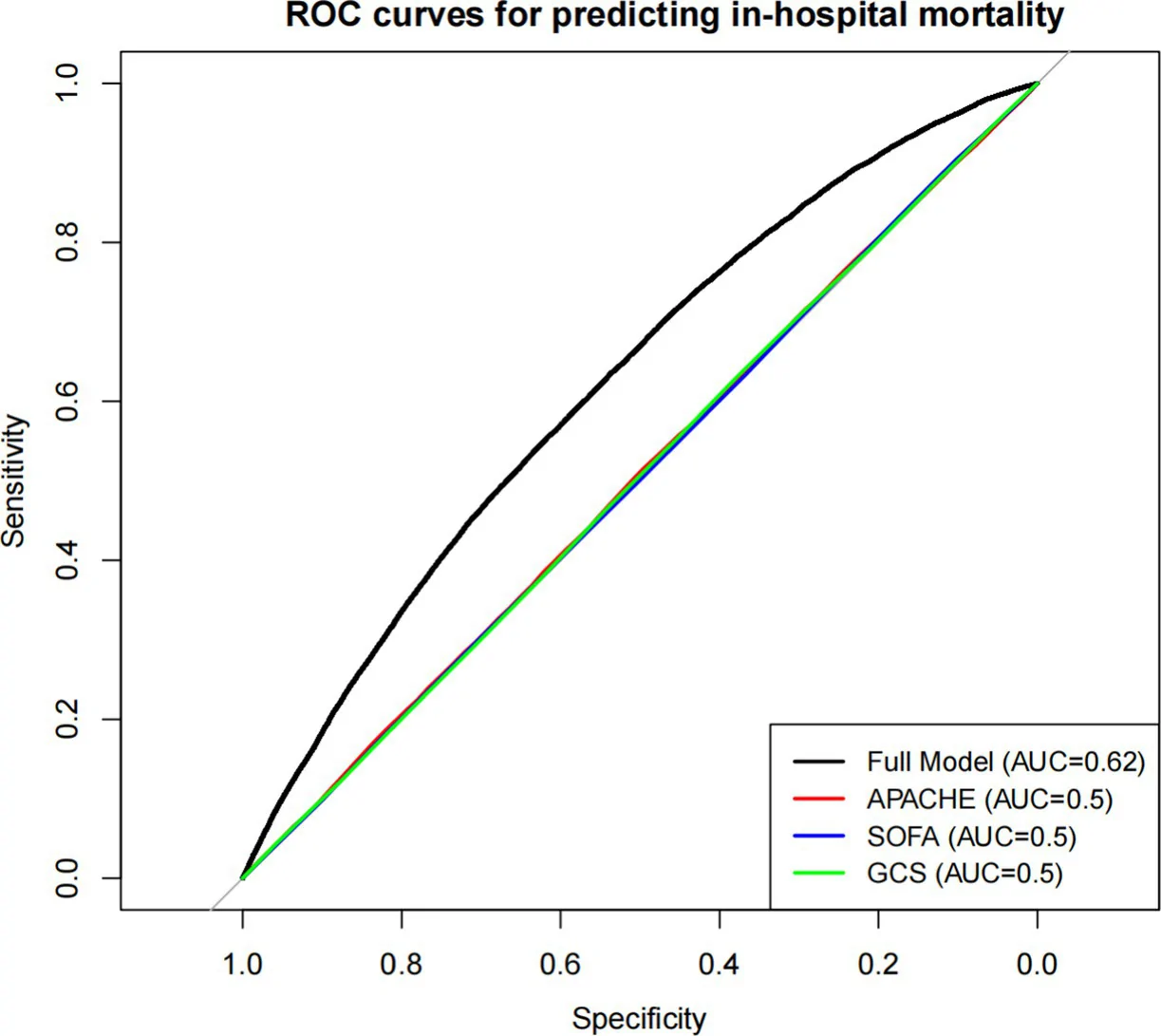
Receiver Operating Characteristic (ROC) curves comparing the predictive performance of the full model with standard clinical scoring systems for 28-Day mortality. This figure evaluates the predictive performance of the developed multivariable comprehensive model (Full Model) compared with individual clinical scoring systems, including APACHE, SOFA, and GCS, for 28-day mortality. The results show that the Full Model achieved an area under the receiver operating characteristic curve (AUC) of 0.62, outperforming the individual APACHE, SOFA, and GCS scores, which demonstrated benchmark AUC values of approximately 0.50 in this specific cohort. Although the individual clinical scores exhibited limited discriminative ability in this analysis, with their ROC curves lying close to the diagonal reference line, the Full Model integrating multiple clinical characteristics demonstrated relatively better predictive performance. These findings suggest that combining multidimensional clinical indicators may provide more comprehensive prognostic information than relying on a single clinical scoring system.

**Figure 6:**
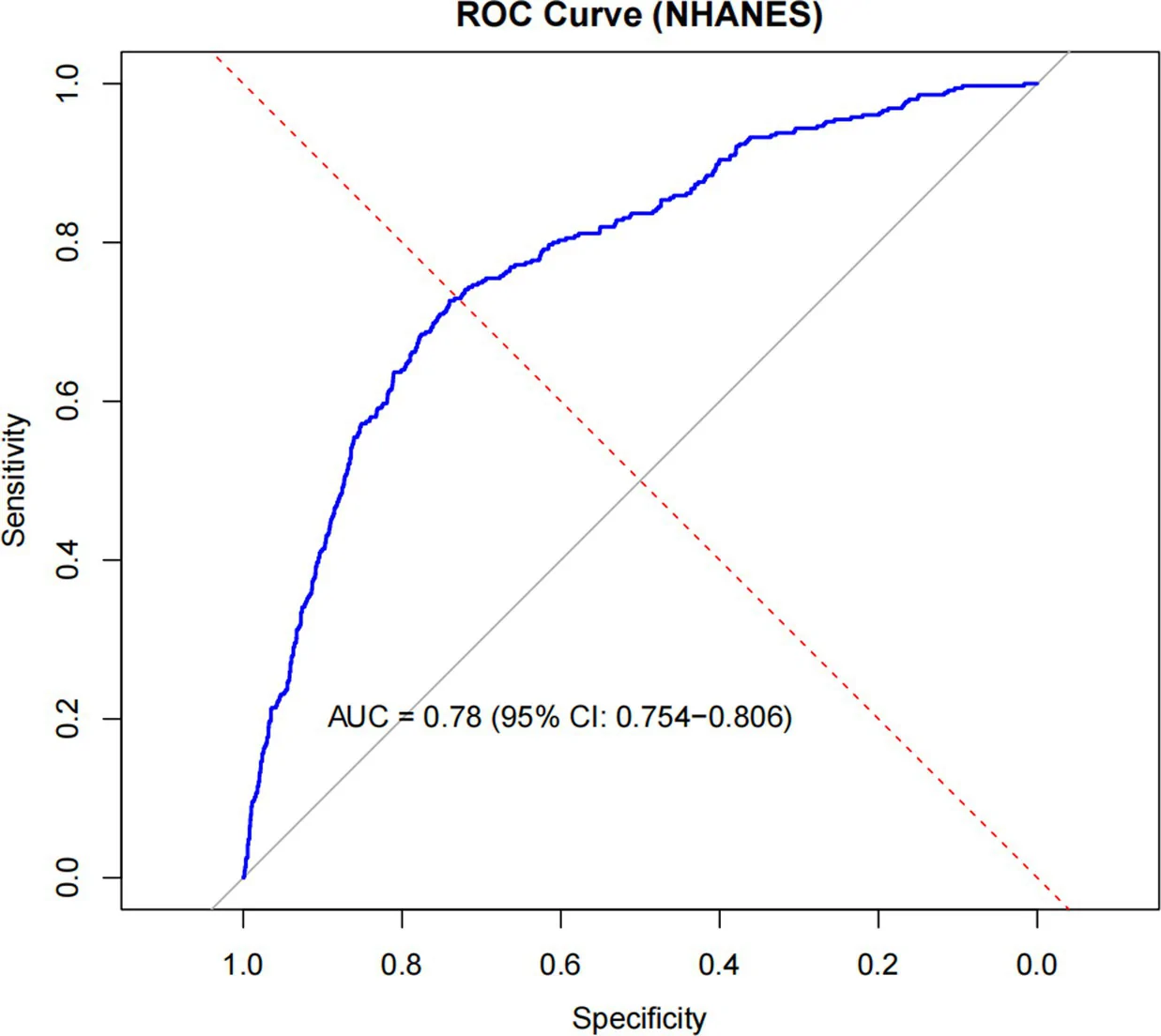
Receiver Operating Characteristic (ROC) curve assessing the discriminative performance of the predictive model. This figure illustrates the overall classification performance of the predictive model developed using the NHANES cohort. The results show that the model achieved an area under the receiver operating characteristic curve (AUC) of 0.78 (95% CI: 0.754–0.806). ③ An AUC between 0.7 and 0.8 indicates good discriminative ability, suggesting that the model can effectively distinguish individuals at higher risk from those at lower risk.

Predictive Model Performance Evaluation:

Discrimination: The area under the receiver operating characteristic curve (AUC) was calculated, and ROC curves were plotted (eICU: Figure 5, NHANES: Figure 6).

**Figure 7:**
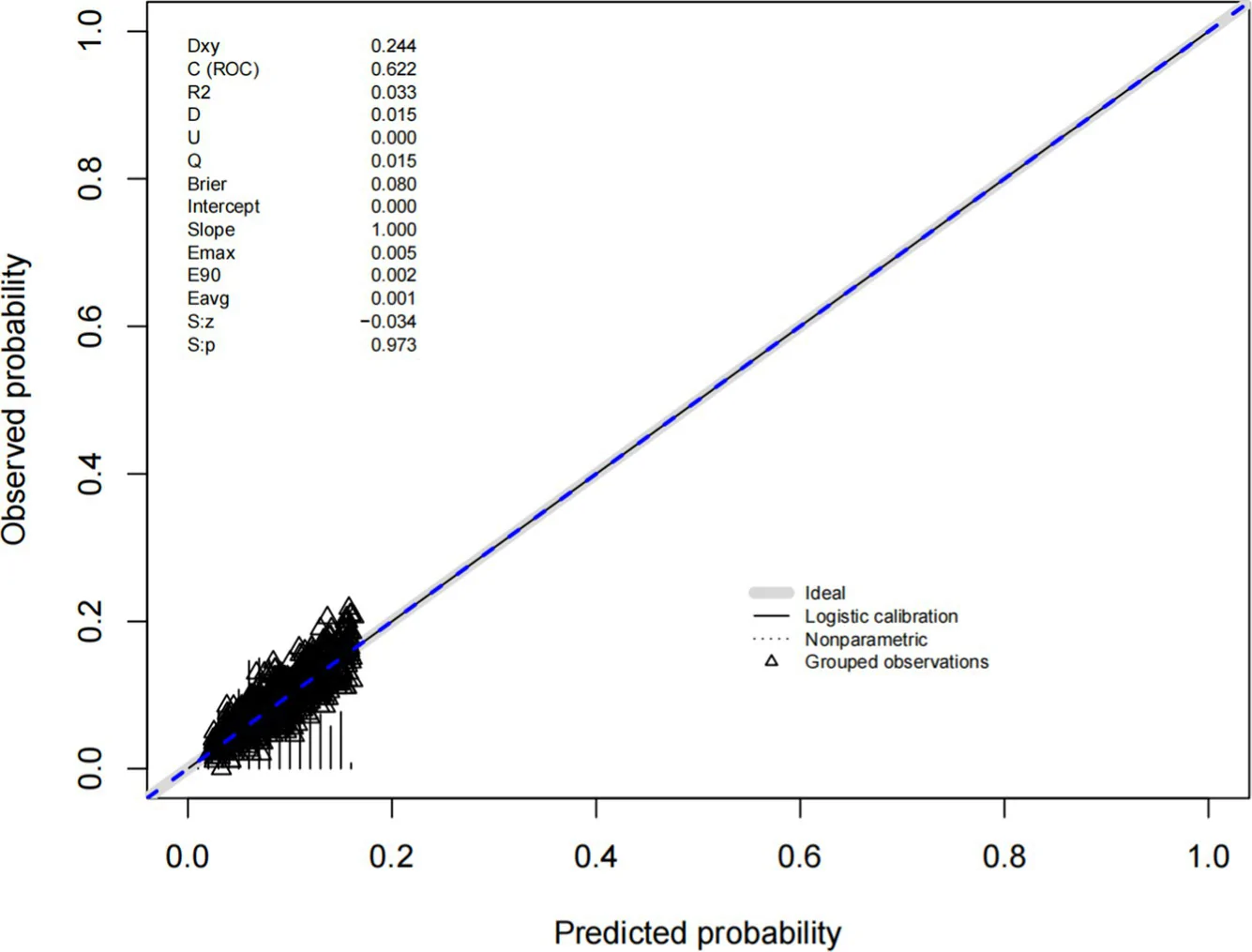
Calibration curve of the predictive model showing the agreement between predicted probabilities and observed outcomes. The calibration curve was used to evaluate the goodness of fit and calibration performance of the predictive model. The blue solid line (Logistic calibration) represents the observed calibration performance of the model, whereas the gray diagonal dashed line (Ideal) represents perfect calibration. The results demonstrate good calibration performance, with a calibration slope of 1.000 and an intercept of 0.000, indicating no apparent systematic overestimation or underestimation of the predicted risk. The Brier score was 0.080, further indicating a relatively small discrepancy between the predicted probabilities and observed outcomes and supporting the overall accuracy of the model’s probability estimates.

**Figure 8:**
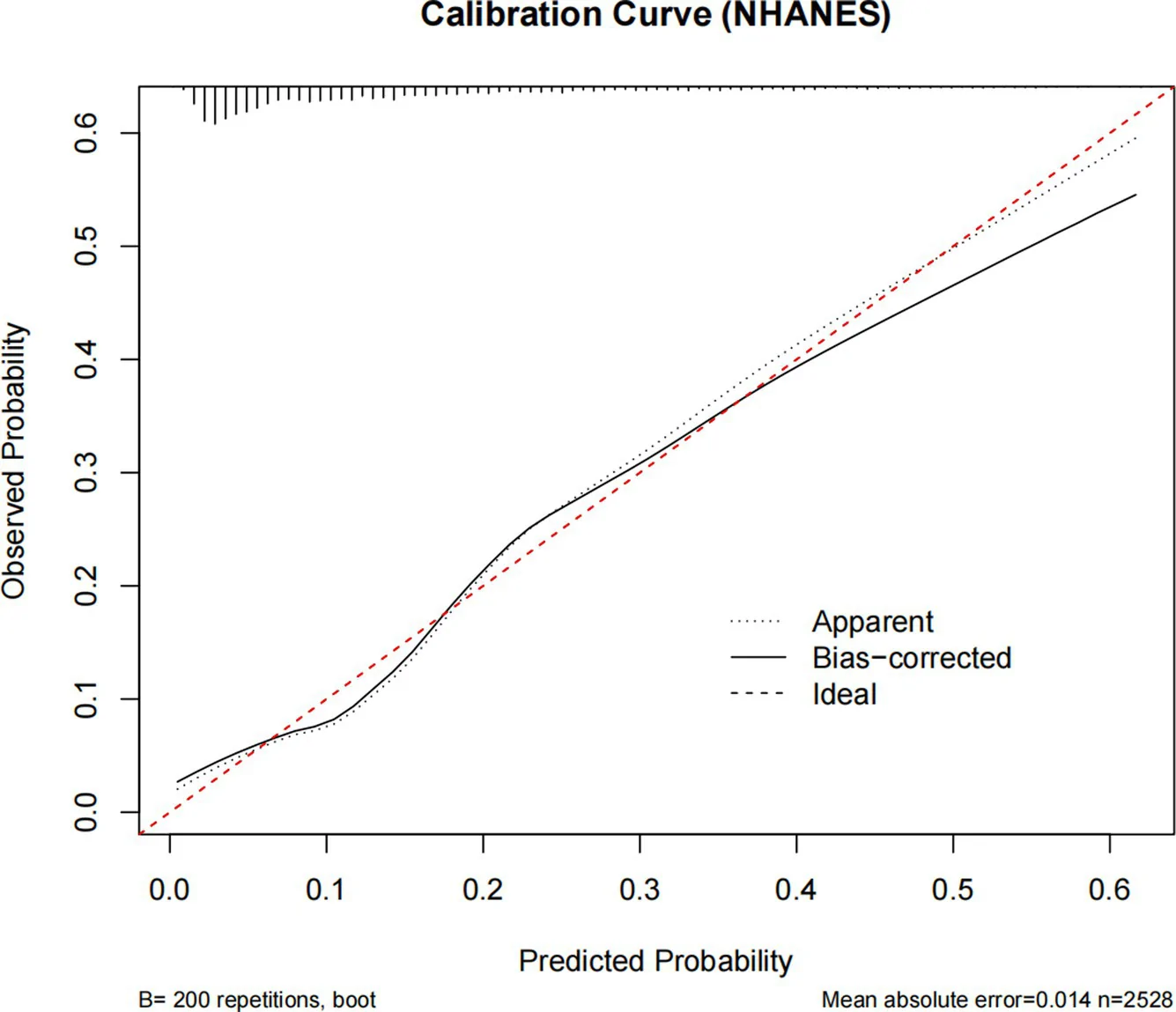
Calibration curve comparing predicted probabilities with observed frequencies using bootstrap validation. The calibration curve was used to assess the accuracy and calibration performance of the predictive model. The dashed line (Ideal) represents perfect calibration, whereas the solid line (Bias-corrected) represents the model’s actual calibration performance after bootstrap correction. The bias-corrected calibration curve closely overlaps with the ideal diagonal line, indicating excellent agreement between the predicted probabilities and the observed event frequencies. The mean absolute error was only 0.014, further quantitatively demonstrating the model’s excellent calibration performance, with no apparent evidence of substantial overfitting or underfitting.

Calibration: Calibration curves were plotted, and internal validation was performed using the bootstrap method (1,000 resamples), calculating the Brier score and mean absolute error (eICU: Figure 7, NHANES: Figure 8). Calibration was assessed primarily using calibration plots and Brier scores where available. Validation was internal only, and no external validation cohort was used. Therefore, model performance should be interpreted cautiously.

**Figure 9:**
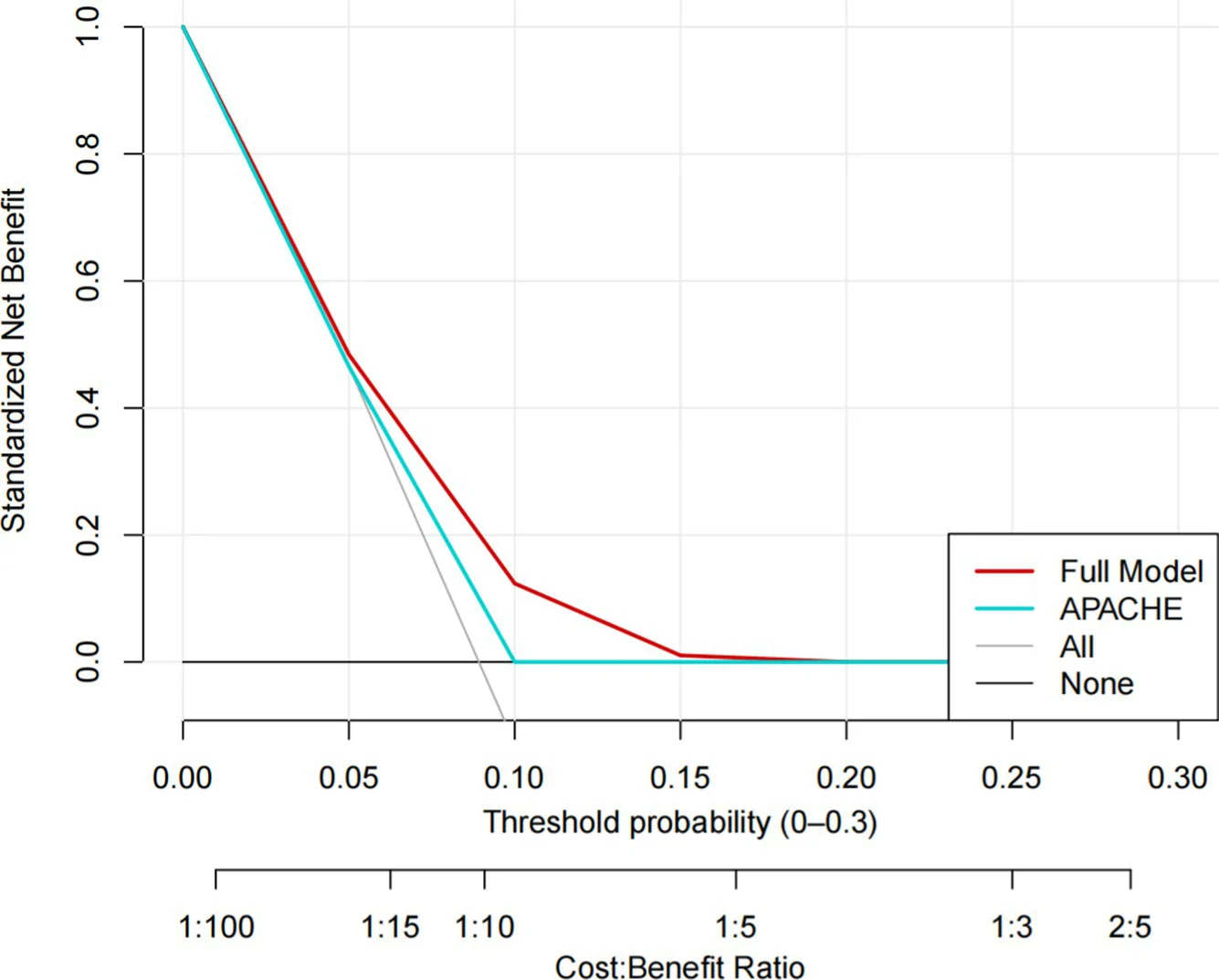
Decision Curve Analysis (DCA) evaluating the clinical net benefit of the predictive model. This figure evaluates the potential clinical utility of the predictive model by comparing the standardized net benefit across different threshold probabilities. The red line represents the Full Model, while the cyan line represents the model based solely on the APACHE score. The gray and black lines represent the “treat-all” and “treat-none” strategies, respectively. ithin the threshold probability range of approximately 0.00–0.15, the Full Model provides a substantially greater net benefit than the APACHE-based model and the other reference strategies. These findings suggest that, at low-to-moderate risk thresholds, using the Full Model to guide clinical decision-making, such as early intervention or resource allocation, may provide greater clinical benefit.

**Figure 10:**
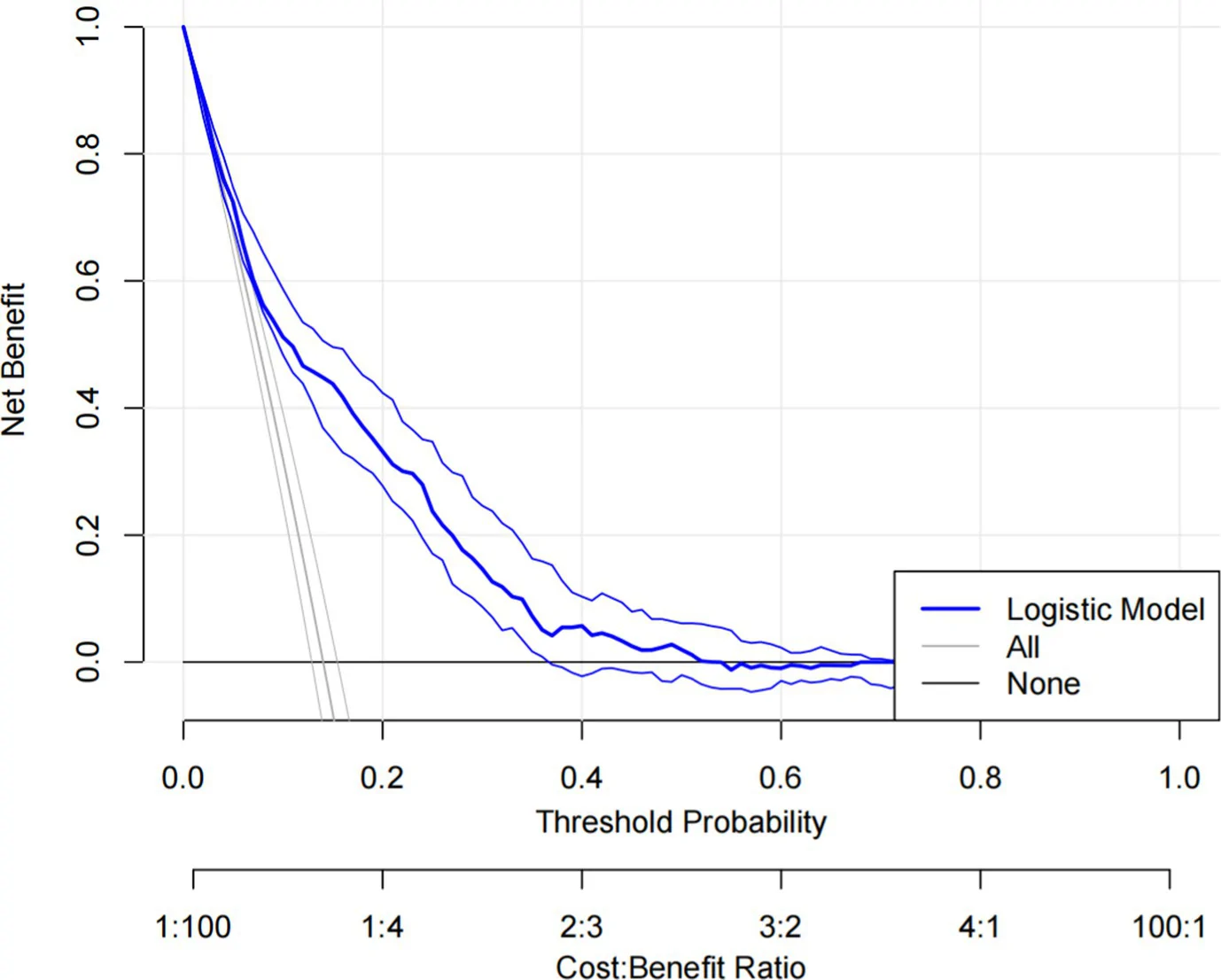
Decision Curve Analysis (DCA) illustrating the clinical net benefit of the logistic model compared with default strategies. Decision curve analysis (DCA) was performed to evaluate the potential clinical utility of the predictive model. The blue solid line represents the logistic regression model, while the gray line and horizontal reference line represent the “treat-all” (All) and “treat-none” (None) strategies, respectively. The results show that across a broad range of threshold probabilities, approximately 0.1–0.7, the logistic model provides a consistently greater net benefit than both reference strategies. These findings suggest that, in clinical practice, using the model for risk stratification and decision-making may provide greater clinical benefit than adopting empirical “treat-all” or “treat-none” strategies.

Clinical Utility: Decision curve analysis (DCA) was performed to assess the clinical net benefit of the models across different decision thresholds (eICU: Figure 9, NHANES: Figure 10).

**Figure 11:**
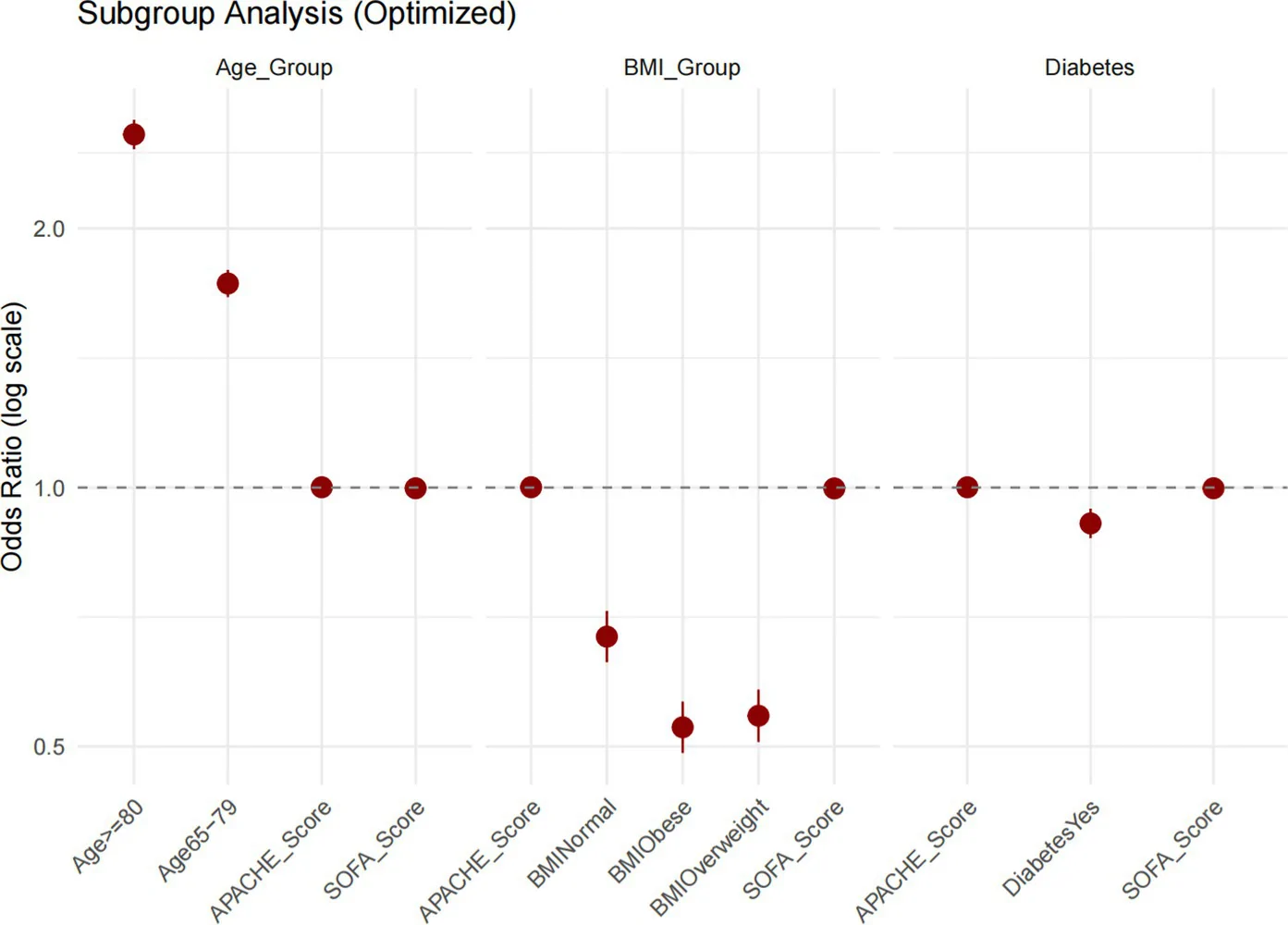
Subgroup analysis of odds ratios for mortality across different demographic characteristics and clinical scores. This figure presents the associations between different subgroups and mortality after stratification of continuous variables, with effect estimates expressed as odds ratios (ORs) on a logarithmic scale. Age effect: The ≥80-year age group (Age ≥80) exhibits the highest OR, substantially higher than that of the 65–79-year group and the reference group, further supporting advanced age as a major risk factor for mortality. Obesity paradox: Compared with the normal-weight group, the obese (BMI Obese) and overweight (BMI Overweight) groups have ORs below 1.0, suggesting that higher BMI may be associated with a lower risk of mortality in this critically ill cohort. This finding is consistent with the phenomenon commonly referred to as the “obesity paradox.” Comorbidities: Patients with diabetes (Diabetes: Yes) also exhibit an OR below 1.0. This association may potentially be related to closer metabolic monitoring in this subgroup or to specific underlying pathophysiological mechanisms.

**Figure 12:**
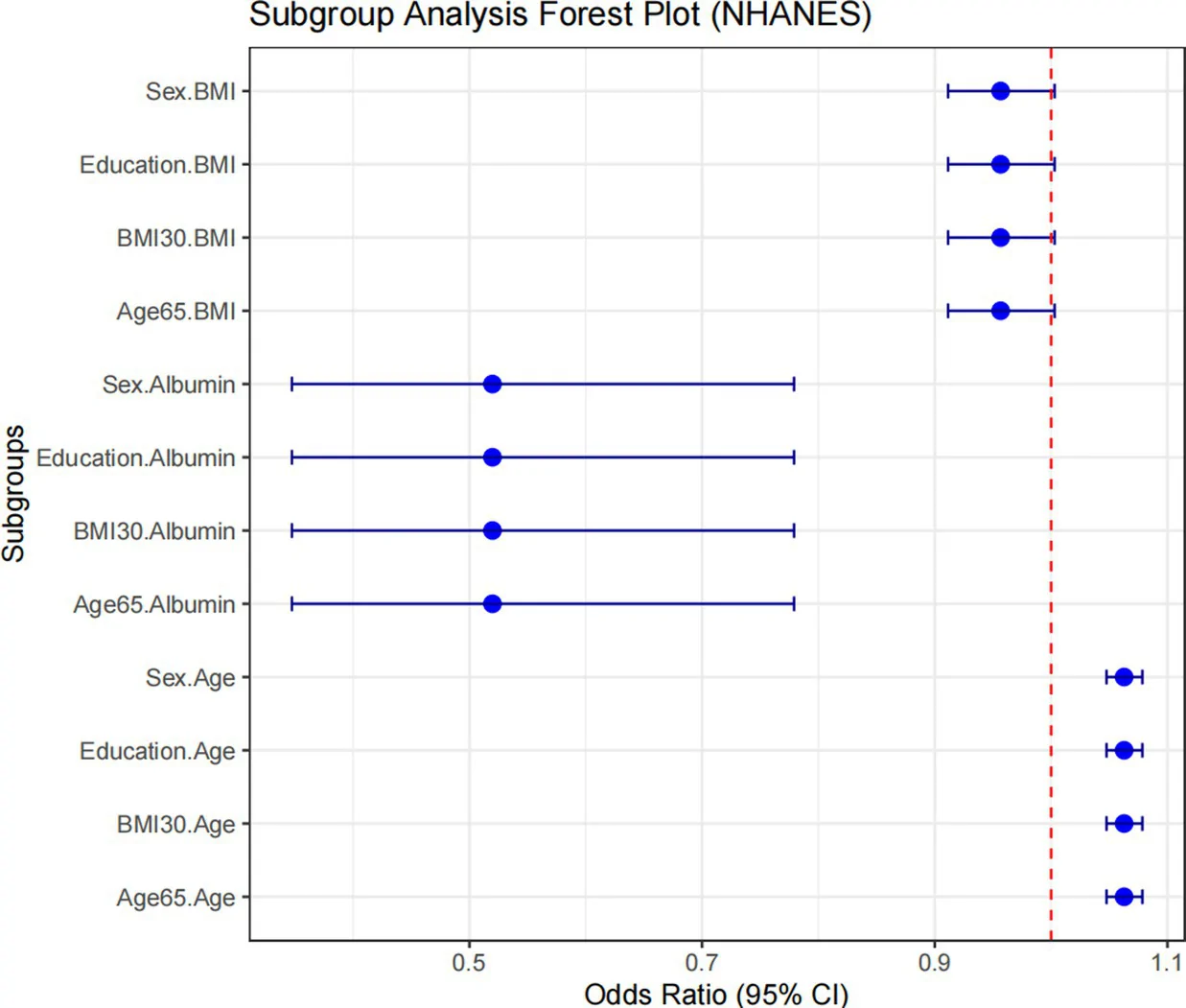
Forest plot of multivariable logistic regression analysis identifying independent predictors associated with the outcome. This figure presents the odds ratios (ORs) and corresponding 95% confidence intervals (CIs) for the associations between individual variables and the outcome in the NHANES cohort. ② Protective factors: Male sex (Gender: Male) shows the most pronounced protective association, with an OR substantially below 1. In addition, higher albumin levels and higher BMI are associated with ORs < 1, suggesting that better nutritional reserves may be associated with more favorable outcomes. Risk factors: Widowed marital status (Marital: Widowed) is associated with a higher OR, suggesting that reduced social support may represent a potential risk factor for the outcome. Other variables: Variables such as vitamin D, glucose, and alcohol consumption (Drinking: Yes) have ORs close to 1, with their 95% CIs crossing the null value of 1.0, indicating that their independent associations with the outcome do not reach statistical significance in this multivariable model.

Subgroup Analysis and Interaction Testing: Subgroup analyses were conducted based on key variables such as age, sex, and BMI. Effect modification was tested by including interaction terms, with results presented as subgroup analysis forest plots (eICU: Figure 11, NHANES: Figure 12).

**Figure 13:**
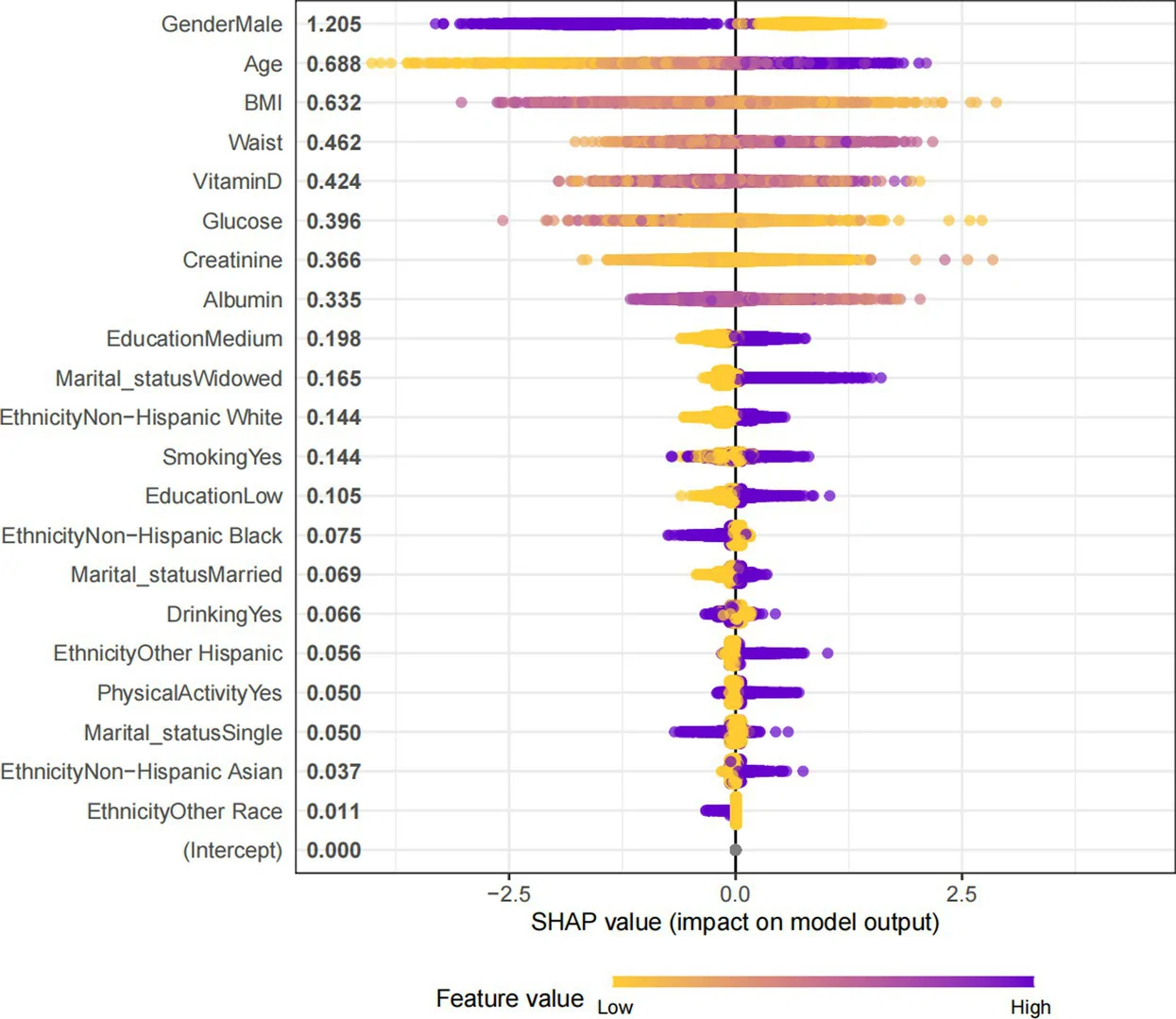
SHAP summary plot ranking the top features contributing to the model output. This figure uses SHAP values to quantify and rank the importance of individual features according to their contributions to the model output. Major contributing features: Sex (GenderMale) is the most influential feature in the model, with a SHAP value of 1.205, followed by age (Age, 0.688) and body mass index (BMI, 0.632). These findings are consistent with the results of the logistic regression analysis, further supporting the important roles of demographic characteristics and anthropometric measures in predicting the study outcome.

Machine Learning and Interpretability Analysis (for NHANES cohort): In the matched NHANES cohort, XGBoost was used as an exploratory model to assess possible nonlinear patterns and variable contributions. Because the matched sample was relatively small, this model was not intended for clinical prediction or deployment. Model performance was evaluated within the analytic sample, and SHAP values were used to describe how individual variables contributed to the model output. SHAP was used only for interpretation, not as a validation method. Therefore, the machine-learning results should be interpreted cautiously and need confirmation in larger independent cohorts (Figure 13).

**Figure 14:**
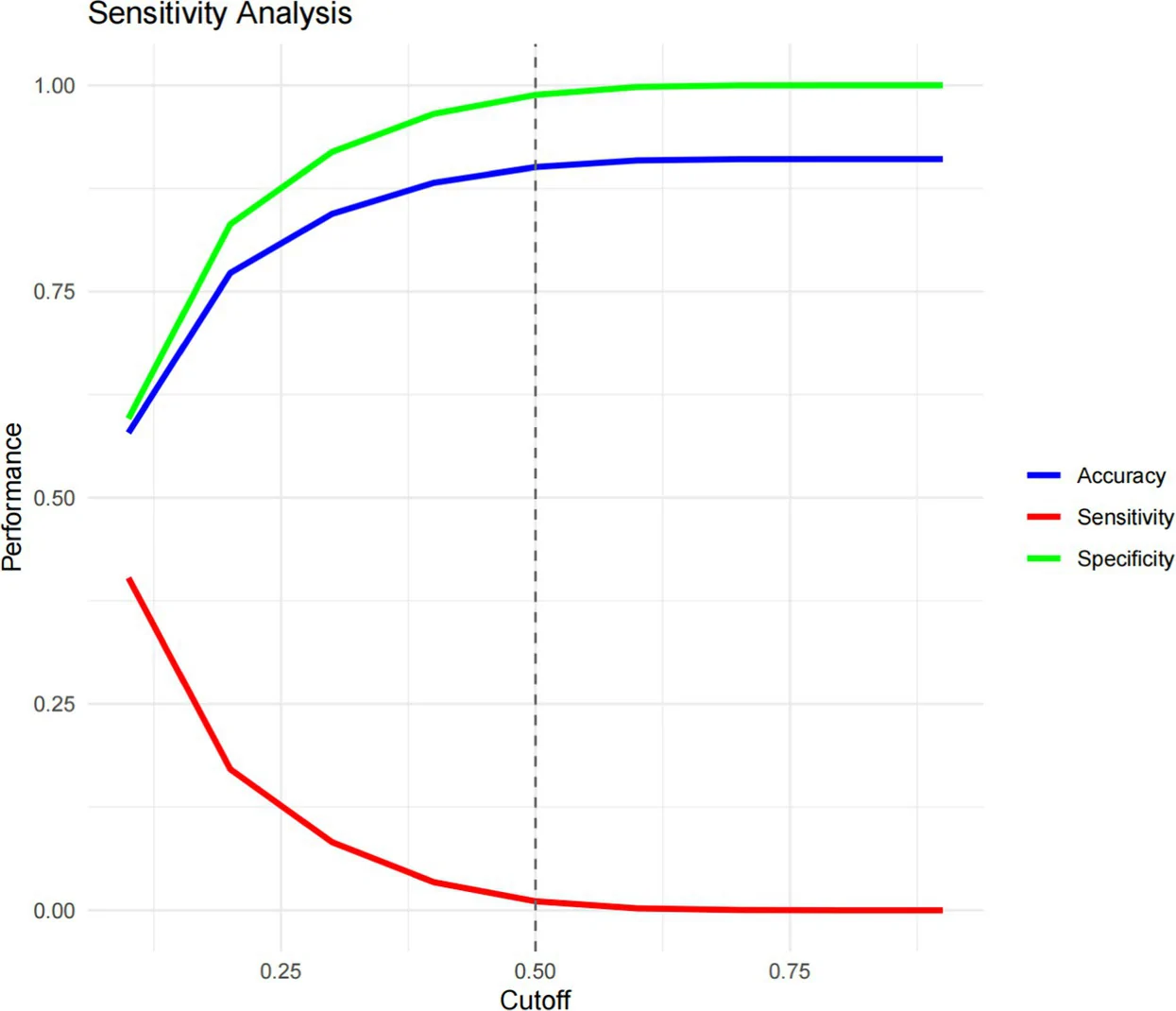
Sensitivity analysis of model performance metrics at different cutoff values.This figure illustrates the dynamic changes in model sensitivity (red line), specificity (green line), and accuracy (blue line) as the classification cutoff value increases from 0 to 1.0. ② Sensitivity decreases rapidly as the cutoff value increases, whereas specificity increases accordingly. The two curves intersect at a cutoff value of approximately 0.10–0.15, suggesting that this range may represent an optimal threshold for balancing the false-negative rate and false-positive rate. ③ The accuracy curve rises rapidly at lower cutoff values and then gradually reaches a plateau, indicating that the model maintains relatively stable overall classification accuracy across a broad range of probability thresholds. This sensitivity analysis can facilitate the selection of an optimal threshold for clinical application according to specific clinical priorities, such as placing greater emphasis on identifying patients with the condition or correctly excluding those without it.

**Figure 15:**
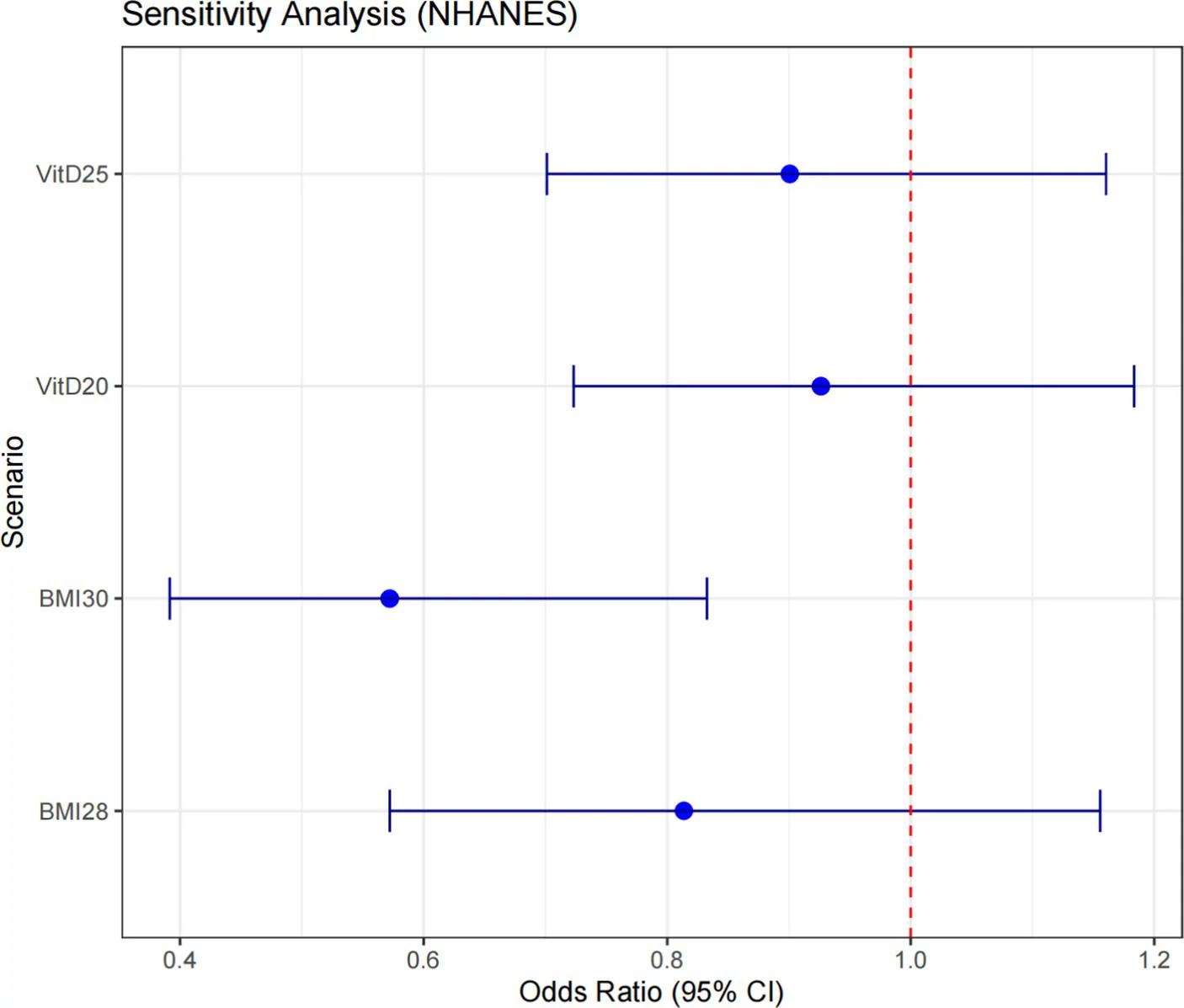
Sensitivity analysis forest plot validating model robustness across different variable. This figure evaluates the robustness of the results by varying the cutoff values or definitions of key variables, such as setting the vitamin D threshold at 20 or 25 and the BMI cutoff at 28 or 30. ② The results show that the direction of the effect estimates remains consistent across different scenarios. In particular, high BMI (BMI ≥ 30) consistently demonstrates a trend toward an OR < 1, although some confidence intervals are relatively wide. These findings suggest that the main results regarding BMI are not dependent on a single specific classification criterion, indicating good robustness of the conclusions.

Sensitivity Analysis: The robustness of the main findings was assessed by altering cutoff values for key continuous variables (e.g., BMI, vitamin D) or incorporating different variable combinations (eICU: Figure 14, NHANES: Figure 15).

Missing data handling: Missing data were examined separately for the NHANES and eICU cohorts before model development. Variables with considerable missingness were assessed based on their clinical importance and suitability for analysis. The main analyses were conducted using complete cases for the variables included in each final model, and multiple imputation was not performed. These results should therefore be interpreted with caution, as missing data may have introduced selection bias.

Clustering consideration for the eICU cohort: The eICU database includes patients from multiple hospitals and ICUs. In this exploratory analysis, hospital-level clustering was not explicitly modeled, and robust standard errors or mixed-effects models were not applied. Therefore, the eICU regression results should be interpreted with caution, as within-hospital correlation may have influenced the estimated standard errors.

**Figure 16:**
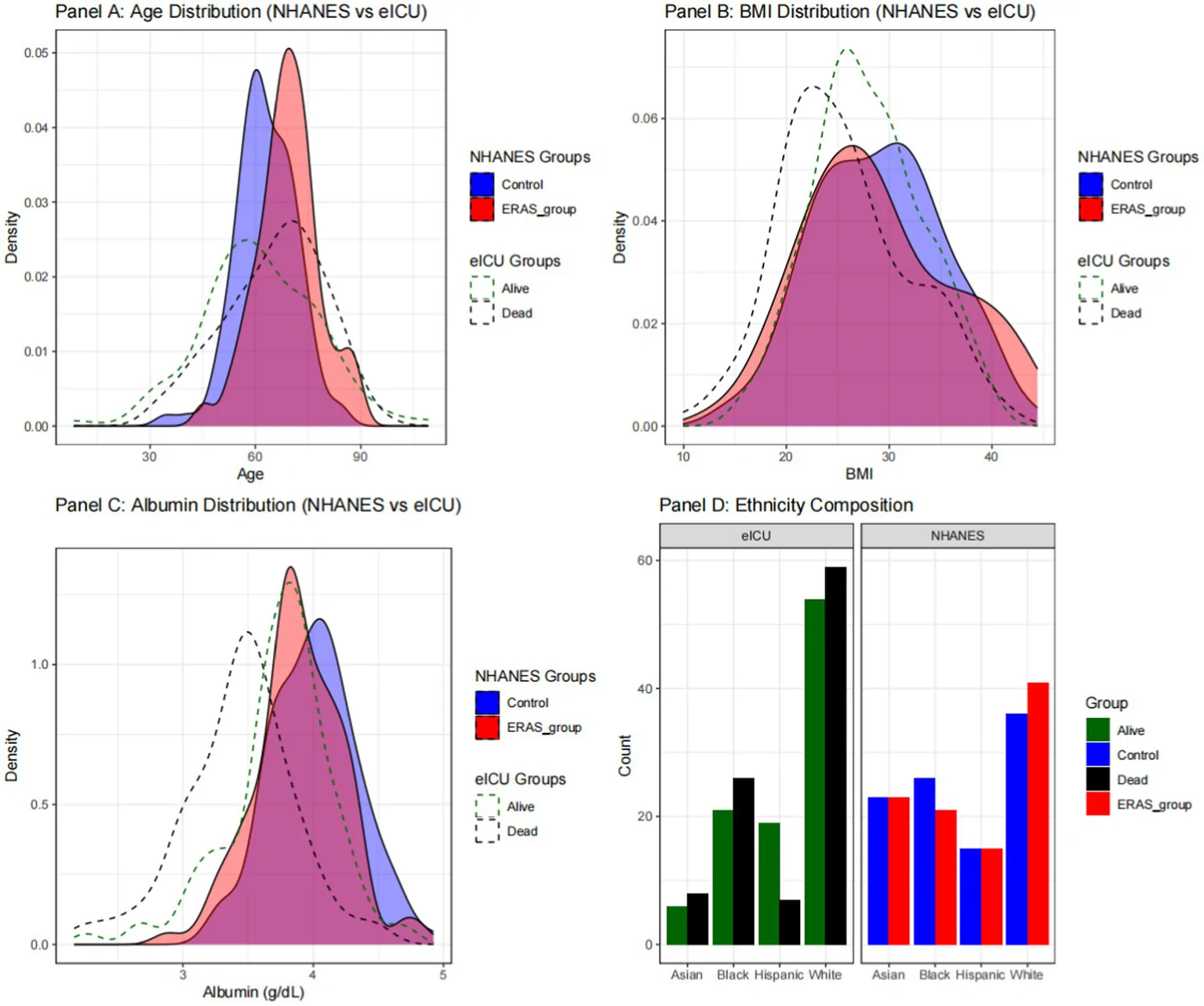
Comparative distribution of demographic and clinical characteristics between the NHANES (General population) and eICU (Critical care) cohorts. Overall comparison: This figure uses four panels (Panels A–D) to visually compare the distributions of key continuous and categorical variables between the NHANES cohort, stratified into the Control and ERAS groups, and the eICU cohort, stratified into the Alive and Dead groups. The figure highlights the baseline heterogeneity between these two independent cohorts. ② Differences in age distribution (Panel A): Panel A presents the density distributions of age. The distributions in the eICU cohort, particularly in the Dead group, are clearly shifted toward older ages, with the highest density concentrated approximately between 60 and 80 years, demonstrating a pronounced aging pattern. In contrast, the age distributions in the NHANES cohort are broader and flatter, covering a wider range of the adult population. This difference is consistent with the clinical characteristics of critically ill populations, who are generally older than the general population. ③ BMI distribution (Panel B): Panel B depicts the density distributions of body mass index (BMI). BMI distributions in both cohorts are approximately normally distributed and are primarily concentrated between 20 and 40 kg/m². Despite the different sources of the two cohorts, their BMI distributions show relatively similar peak locations, suggesting that overweight and obesity are common characteristics in both populations. However, the eICU cohort appears to have a longer distributional tail, potentially reflecting the presence of more extreme BMI values among critically ill patients. ④ Marked differences in albumin levels (Panel C): Panel C focuses on serum albumin levels, which represent one of the most prominent features distinguishing the health profiles of the two cohorts. The NHANES cohort, representing a relatively healthier general population, shows a tall and narrow albumin distribution concentrated around the normal-to-high range (approximately 4.0 g/dL). In contrast, the eICU cohort, representing critically ill patients, exhibits a markedly left-shifted and broader distribution, with the peak primarily between 2.5 and 3.0 g/dL. This pronounced separation in albumin distributions may reflect the effects of nutritional depletion, systemic inflammation, and acute illness among critically ill patients. ⑤ Comparison of racial composition (Panel D): Panel D uses bar charts to compare the racial composition of the two databases. Both cohorts include Asian, Black, Hispanic, and White participants. White participants constitute the largest racial group in both eICU and NHANES; however, the proportions of racial and ethnic minority groups differ between the two databases. These differences may be attributable, at least in part, to geographic variation in data collection and differences in the populations served by participating hospitals and study centers.

Cross-Database Comparison: Combined distribution plots were created for key continuous variables (age, BMI, serum albumin) and categorical variables (race; Figure 16), providing a visual comparison of systematic differences in demographic and clinical characteristics between the two study cohorts.

## 3 Results

### 3.1 Baseline characteristics of the study populations

Baseline characteristics of the eICU orthopedic critical care cohort are shown in (Table 2). Compared to the survivor group, the non-survivor group was significantly older (68.19 vs. 61.79 years, *p* < 0.001), had greater illness severity (APACHE IV score: 97.50 vs. 59.43; SOFA score: 6.38 vs. 3.25), and a significantly higher proportion received invasive mechanical ventilation (56.8% vs. 25.1%). Serum albumin levels were significantly lower in the non-survivor group (2.49 vs. 2.92 g/dL, *p* < 0.001).

Baseline characteristics of the NHANES community orthopedic cohort before matching are shown in Table 1. The ERAS-inclined group showed highly significant differences from the control group across multiple dimensions, including age (69.05 vs. 64.15 years), sex composition (female 81.1% vs. 44.9%), and history of fracture (89.3% vs. 0%), confirming severe selection bias. After PSM, 710 participants (355 in each group) entered the final analysis. The Love Plot (Figure 1) showed that SMDs for all covariates converged to within 0.1, indicating successful matching and good baseline balance between groups.

### 3.2 eICU cohort: determinants of prognosis in the critical phase

In the eICU cohort, older patients, particularly those aged ≥80 years, had poorer observed in-hospital survival (Figure 2). In adjusted logistic regression, mechanical ventilation was associated with increased in-hospital mortality, while higher serum albumin levels were associated with lower mortality risk (Figure 3). Given the different measurement scales of these predictors, their odds ratios should not be compared directly. A model incorporating age, mechanical ventilation, and albumin showed better discrimination than the APACHE IV score alone (AUC = 0.62, Figure 5).

### 3.3 NHANES cohort: determinants of long-term rehabilitation outcomes

In the matched NHANES cohort, multivariable logistic regression analysis (Figure 4) identified the following independent predictors:

Protective Factors: Male sex (OR = 0.45, 95% CI: 0.33–0.61) and higher serum albumin level (OR = 0.85 per 1 g/dL increase) were associated with better long-term rehabilitation outcomes.

Risk Factor: Widowed status (OR = 1.52, 95% CI: 1.05–2.20) indicated an increased risk of poor outcomes.

**Figure 17:**
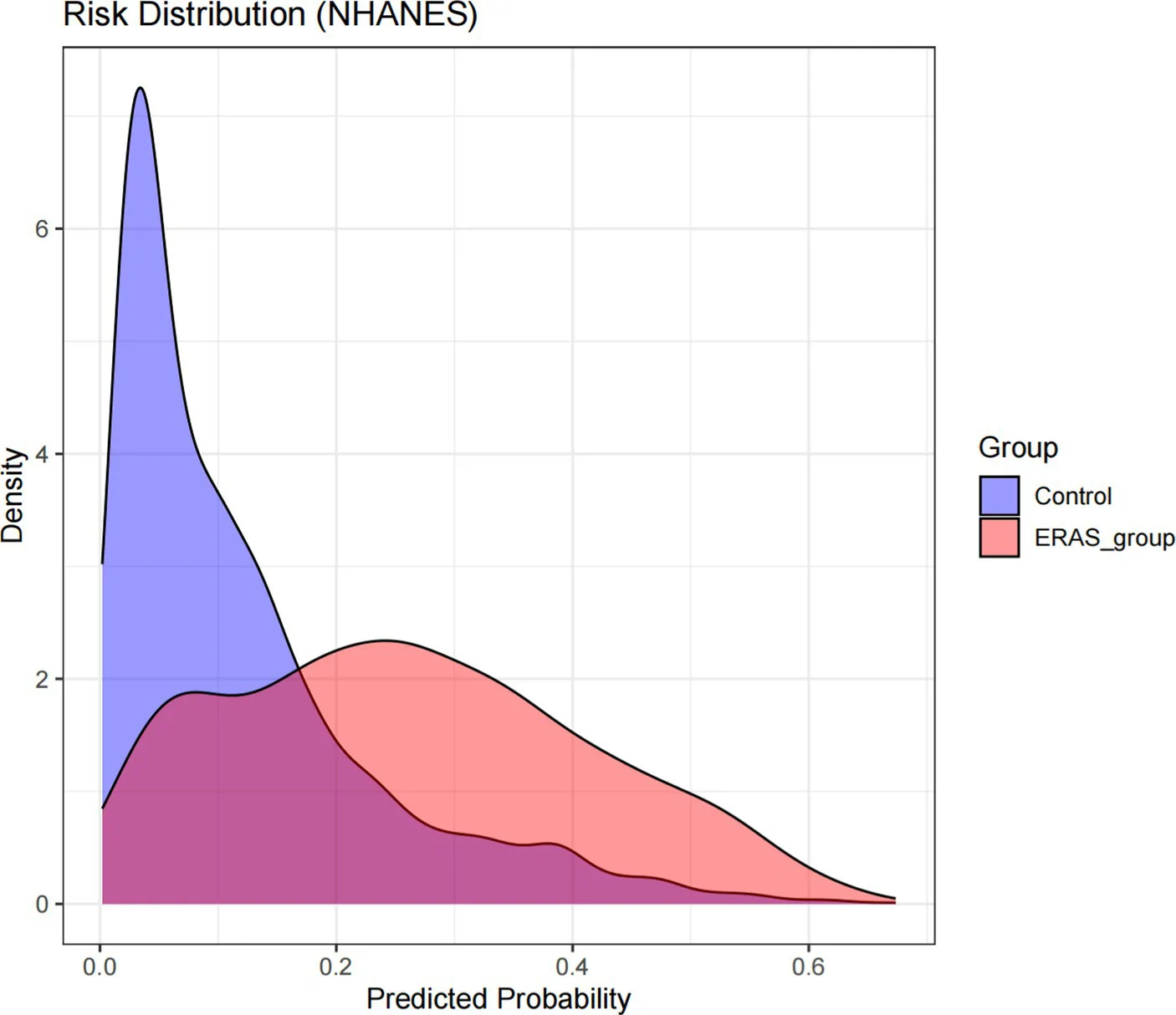
Density plot Comparing the distribution of predicted probabilities between the control and ERAS groups. This figure illustrates the distributions of model-predicted probabilities in the two study groups. Control group (blue): The distribution is markedly right-skewed, with a pronounced peak concentrated in the low-probability range (0.0–0.1), indicating that most individuals in the control group have relatively low predicted probabilities. ERAS group (red): The distribution is flatter and broader, with the peak shifted toward the 0.2–0.3 probability range and a long tail extending toward higher predicted probabilities. The clear separation between the two probability distributions visually demonstrates the model’s ability to discriminate between the risk profiles of the Control and ERAS groups.

BMI showed a weak protective trend (OR = 0.97, 95% CI: 0.95–0.99). The prediction model based on these variables demonstrated excellent performance: discrimination AUC was 0.78 (95% CI: 0.754–0.806, Figure 6), and calibration was excellent (mean absolute error = 0.014, Figure 8). Decision curve analysis (Figure 10) confirmed that using this model to guide decisions provided significant clinical net benefit across a broad threshold probability range of 0.1 to 0.7. SHAP analysis suggested that sex, BMI, age, and albumin contributed notably to the XGBoost model output. These findings indicate that both physiological reserve and sociodemographic factors may be related to postoperative recovery patterns. However, the SHAP results should be interpreted as model-based explanations rather than independent validation, particularly for the observed contribution of sex (Figures 13, 17).

### 3.4 Cross-database comparison of serum albumin distribution

The combined distribution plots (Figure 16) visually contrast the heterogeneity of the two cohorts. Panel A shows that eICU patients (especially non-survivors) were more concentrated in older age groups. Panel B shows similar BMI distribution shapes between cohorts, but the eICU cohort distribution was more dispersed. The most distinctive finding is shown in Panel C for serum albumin distribution: the NHANES population’s albumin exhibited a tall, thin peak centered in the normal-high range (~4.0 g/dL); whereas the eICU population’s distribution was overall shifted left and flattened, with its peak in the low range (2.5–3.0 g/dL). The observed leftward shift in albumin distribution is consistent with greater illness severity and nutritional depletion in the eICU cohort compared with the NHANES community cohort. Together with the regression findings, this pattern supports serum albumin as a potentially useful prognostic correlate across settings. However, the distributional comparison is descriptive and does not by itself establish albumin as a universal biomarker.

## 4 Discussion

This study, through the innovative parallel analysis of the NHANES and eICU databases, constructs a comprehensive analytical framework spanning from community long-term health baseline to in-hospital acute critical status. It systematically reveals the multi-level, context-dependent spectrum of factors influencing outcomes in orthopedic surgical patients. The main findings can be summarized as a dual-driver model: a patient’s short-term survival crisis is primarily determined by the intensity and cost of perioperative acute physiological insult; whereas long-term functional recovery is more profoundly constrained by their preoperatively accumulated “health capital” and psychosocial structure. The key biological bridge connecting these two settings is the nutritional and inflammatory homeostasis represented by serum albumin.

### 4.1 Serum albumin as a prognostic correlate in perioperative risk assessment

Serum albumin showed a relatively stable association with outcomes in both cohorts. In the eICU cohort, patients with lower albumin levels, especially those near or below 2.5 g/dL, tended to have worse survival outcomes. In the NHANES cohort, higher albumin levels within the normal range were also linked to more favorable long-term recovery. These findings are clinically plausible, as albumin reflects nutritional status as well as inflammation-related physiological changes. Beyond its role as a nutritional marker, albumin contributes to inflammatory regulation and capillary integrity (22). After surgical trauma, inflammatory responses may suppress albumin synthesis, accelerate catabolism, and increase extravascular leakage, resulting in a decline in circulating albumin levels (23). After surgical trauma, inflammatory responses may suppress albumin synthesis, accelerate catabolism, and increase extravascular leakage, resulting in a decline in circulating albumin levels. Therefore, in the context of ERAS care, low albumin should mainly be used to identify patients who may require closer nutritional evaluation, inflammatory assessment, and prehabilitation support. This study does not demonstrate that albumin correction itself improves outcomes.

### 4.2 The “vulnerability paradox” of ERAS efficacy and pathway optimization

The ERAS-inclined group in the NHANES cohort had greater baseline vulnerability before matching, as shown in Table 1. This finding suggests a possible “vulnerability paradox” in ERAS practice: patients who are older, have more comorbidities, or have poorer functional status may be more likely to require enhanced perioperative support, but their limited physiological reserve may also make it difficult for them to follow or benefit equally from standard ERAS measures, such as early ambulation. Albumin and BMI should probably be interpreted with caution. In this study, they may not simply represent nutritional status, but may also reflect frailty and overall physiological reserve to some extent. When considered together with age and marital status, these findings suggest that recovery after orthopedic surgery is influenced by a broader set of patient vulnerabilities, including nutritional condition, muscle reserve, mobility, cognitive status, and available social support. The poorer outcomes observed among widowed patients may be partly related to the lack of daily caregiving, lower adherence to rehabilitation plans, social isolation, or greater psychological stress, which could all affect long-term functional recovery. These findings indicate that ERAS pathways may need to be adapted according to patients’ baseline risk rather than applied as a uniform protocol. For older or vulnerable patients, assessment of nutrition, physical function, cognition, psychological status, and social support may help guide a more individualized perioperative plan. The form of vulnerability may also vary by orthopedic procedure. For patients undergoing elective arthroplasty, frailty may mainly limit early mobilization and participation in functional rehabilitation. For fracture or trauma patients, acute injury, pain, delirium risk, and the lack of sufficient preoperative optimization may make standard ERAS components more difficult to apply. In spine surgery, preoperative neurological status, pain severity, and mobility limitation may also affect recovery. These differences suggest that frail older adults, patients with cognitive vulnerability, and those with limited social support may benefit from a more gradual rehabilitation plan, closer nutritional and functional assessment, and discharge planning tailored to their actual level of support. For vulnerable patients at higher risk, ERAS may need to be delivered more gradually, with stronger perioperative support and earlier involvement of a multidisciplinary team, such as dietitians, physiotherapists, and social workers. The association between male sex and the ERAS-inclined recovery profile should be interpreted with caution. It may not reflect a direct biological protective effect, but rather residual confounding or differences in case mix. Unmeasured factors, such as baseline functional status, type of surgery, postoperative support, and access to rehabilitation, may also have contributed to this result.

### 4.3 Revisiting the “obesity paradox” and body composition management

A higher BMI or obesity classification was associated with more favorable outcomes in both cohorts (Figures 4, 11, 12), a pattern often described as the “obesity paradox.” This finding should be interpreted cautiously. In critically ill patients, a higher BMI may partly reflect greater energy reserve during acute stress, although BMI alone cannot distinguish fat mass from muscle mass. In the community recovery setting, higher BMI may also be related to better metabolic reserve or preserved lean body mass in some patients. Therefore, preoperative optimization in orthopedic surgery should not focus only on weight reduction, but should also consider body composition. Preserving or improving muscle mass through nutritional support and resistance-based rehabilitation may be more meaningful than BMI reduction alone. Combining BMI with functional or muscle-related measures, such as grip strength or calf circumference, may provide a more accurate assessment of physiological reserve (24).

### 4.4 Methodological significance and clinical translation

This study shows that publicly available databases can be useful for examining orthopedic recovery from different clinical settings. The prediction models developed here are not accurate enough for direct individual-level bedside decision-making, but the identified factors, such as mechanical ventilation, low albumin, and widowed status, may be useful candidates for future early-warning models in ICU and ward settings (25–27). For example, for orthopedic patients transferred to the ICU postoperatively requiring mechanical ventilation and with hypoalbuminemia, the highest level of monitoring and support should be initiated (28). Decision curve analysis (Figures 9, 10) quantifies the potential benefit of using a data-driven model to aid decision-making compared to empirical strategies, providing economic and utility evidence for clinical practice.

### 4.5 Study limitations

This study has several limitations. First, its observational nature limits causal inference (29). Second, the ERAS grouping in NHANES relied on self-report, which may be subject to recall bias and misclassification (30). Third, the eICU database lacks orthopedic-specific functional outcomes (e.g., range of motion, walking ability) and detailed records of ERAS protocol adherence. Finally, the populations and temporal contexts of the two databases differ; we did not perform individual-level data linkage or meta-analysis but adopted a comparative interpretation strategy (29, 31). In addition, the eICU database provides reliable in-hospital outcome information but does not allow consistent assessment of post-discharge 28-day or 180-day mortality. Therefore, the eICU analyses were interpreted as associations with in-hospital mortality rather than long-term survival. Hospital-level clustering in the multicenter eICU database was not explicitly modeled, which may have affected standard error estimates and the precision of statistical inference. Several limitations should be noted. The ERAS-inclined recovery profile in NHANES was based on self-reported postoperative recovery characteristics rather than prospectively recorded ERAS adherence, so exposure misclassification is possible. Although propensity score matching was used, residual confounding cannot be excluded, particularly because the two groups showed limited clinical overlap and clear case-mix differences. Outcome definitions also differed between databases, and the NHANES recovery-related outcome may not fully represent standardized orthopedic functional recovery. In addition, direct frailty measures, procedure-specific information, and detailed ERAS protocol adherence were not available (32). Missing data were handled by complete-case analysis, and NHANES survey design features were not incorporated, which may limit generalizability. Finally, the prediction models were internally evaluated only and should be externally validated before any clinical application.

## 5 Conclusion

By combining data from a community health survey and a critical care database, this study provides a staged understanding of factors related to recovery outcomes in orthopedic patients. The findings suggest that serum albumin may serve as an important link between baseline health reserve and tolerance to postoperative physiological stress, highlighting the value of perioperative nutritional monitoring. In addition, the results point to a potential “vulnerability paradox” in ERAS practice: patients who are older, nutritionally compromised, or socially unsupported may require more individualized and supportive ERAS strategies rather than a uniform pathway.

Based on the above findings, we propose that orthopedic ERAS pathways may inform future development of individualized perioperative risk-stratification strategies. In the preoperative phase, routine screening for nutritional risk (focusing on albumin assessment) and social vulnerability should be conducted, coupled with prehabilitation. During the intraoperative and immediate postoperative acute phase, efforts should focus on minimizing physiological stress, optimizing analgesia, and promptly identifying patients with severe respiratory failure or high acute illness burden, for whom mechanical ventilation may serve as a marker of clinical severity rather than a directly modifiable ERAS target. In clinical practice, perioperative albumin assessment and nutritional risk screening may help identify patients who need closer monitoring and more individualized perioperative support within ERAS pathways. Future studies should test these findings in larger multicenter orthopedic cohorts. They should also include patient-reported outcomes, changes in functional recovery over time, and repeated biomarker measurements, so that perioperative risk assessment can better reflect the patient’s actual recovery process.

## Data Availability

The datasets presented in this study can be found in online repositories. The names of the repository/repositories and accession number(s) can be found in the article/supplementary material.
